# Open Data In Neurophysiology: Advancements, Solutions & Challenges

**Published:** 2024-07-01

**Authors:** Colleen J. Gillon, Cody Baker, Ryan Ly, Edoardo Balzani, Bingni W. Brunton, Manuel Schottdorf, Satrajit Ghosh, Nima Dehghani

**Affiliations:** 1) Department of Bioengineering, Imperial College London, London, UK.; 2) CatalystNeuro, Benicia, CA, USA.; 3) Scientific Data Division, Lawrence Berkeley National Laboratory, Berkeley, CA, USA.; 4) Center for Computational Neuroscience, Flatiron Institute, New York, NY, USA.; 5) Department of Biology, University of Washington, Seattle, WA, USA.; 6) Princeton Neuroscience Institute, Princeton University, Princeton, NJ, USA.; 7) McGovern Institute for Brain Research, MIT, Cambridge, MA, USA.; 8) † These authors contributed equally to this paper.

## Abstract

Across the life sciences, an ongoing effort over the last 50 years has made data and methods more reproducible and transparent. This openness has led to transformative insights and vastly accelerated scientific progress^1,2^. For example, structural biology^3^ and genomics^4,5^ have undertaken systematic collection and publication of protein sequences and structures over the past half-century, and these data have led to scientific breakthroughs that were unthinkable when data collection first began (e.g.^6^). We believe that neuroscience is poised to follow the same path, and that principles of open data and open science will transform our understanding of the nervous system in ways that are impossible to predict at the moment.

To this end, new social structures along with active and open scientific communities are essential^7^ to facilitate and expand the still limited adoption of open science practices in our field^8^. Unified by shared values of openness, we set out to organize a symposium for Open Data in Neuroscience (ODIN) to strengthen our community and facilitate transformative neuroscience research at large. In this report, we share what we learned during this first ODIN event. We also lay out plans for how to grow this movement, document emerging conversations, and propose a path toward a better and more transparent science of tomorrow.

## I. OPEN DATA IN NEUROSCIENCE

The symposium for Open Data in Neuroscience (ODIN) 2023, hosted by the initiative of the same name under the auspices of the McGovern Institute for Brain Research at the Massachusetts Institute of Technology, assembled experts in the development of advanced tools, methods, and models in neurophysiology. Exploring recent and forthcoming advancements in neurophysiology, the group paid particular attention to the increased resolution of emerging technologies and anticipated growth of data over the next years. Thus, a focus of the dialogue were challenges these technologies are expected to present for existing data infrastructures and for the broader adoption of open science practices. The symposium sought to foster collaborative discourse identifying such challenges, as well as mitigation strategies and solutions. Crucially, these technological advancements mark a significant frontier in neurophysiological research. There is a clear imperative for novel mathematical and computational models, and artificial intelligence (AI) or machine learning (ML) solutions that will enable the community to effectively navigate and leverage the full potential of high-resolution, high-dimensional, and multimodal data.

**FIG. 1:**
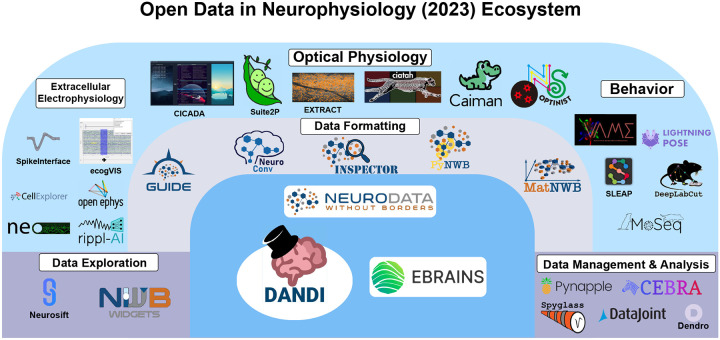
The ecosystem of open source neurophysiology toolkits presented or discussed during ODIN 2023. See Table I for more information about each toolkit.

The ODIN symposium was structured in a manner reminiscent of the Brain Research through Advancing Innovative Neurotechnologies (BRAIN) Initiative meetings and COSYNE (Computational & System Neuroscience Meeting) as single stream sessions. The symposium’s agenda was organized as a series of succinct presentations on themes ranging from acquisition devices to simulated neural activity models (Fig. 1). The latest large-scale open releases of neurophysiology data and novel insights derived from these datasets were also discussed. The selection of presenters was intended to showcase the neurophysiology community’s various subfields and a diversity of perspectives, covering a spectrum of emerging scholars to established scientists. Each session of talks was followed by an interactive discussion between the audience and presenters. Additionally, each day’s topics were revisited in an hour long synthesis session that focused on common themes and their connections to one another.

The symposium’s agenda was organized into a series of sessions over three days, each addressing critical facets of neurophysiology research:

- **Day 1: Devices, Neuroinformatics, and Platforms**, featuring sessions on new devices and high throughput acquisition systems, the neuroinformatics of neurophysiology, and platforms/infrastructures that underpin research efforts.
- **Day 2: Knowledge Extraction, Software, Modeling**, encompassing discussions on OpenData2Knowledge pipelines for deriving scientific insights from multi-scale, high-dimensional data, neuroscience toolkits promoting open software and science, and approaches to modeling and benchmarking.
- **Day 3: Neuroinformatics Breakouts**. In the final day of the meeting, the audience and presenters broke up into small discussion groups tasked with (1) delving in greater detail into the problems faced by neurophysiology as a field, and proposed solutions, including common infrastructure, data formats and standards, and (2) exploring the role of AI/ML, computing, and visualization in enhancing neuroinformatics.

Overall, ODIN 2023 was characterized by palpable enthusiasm and rich exchanges, reflecting the community’s need for a commitment to advancing open science in neuroscience. To sustain this momentum, we aim to continue this symposium as a bi-annual event. This continuity will reinforce the symposium’s founding principles of open communication and collaborative exploration across diverse neurophysiology and systems neuroscience domains. In this paper, we present a comprehensive overview of ODIN 2023. Our aim is to enable and inspire the broader neurophysiology community to join us in this project. We begin by providing concise summaries of each session and discussion (with full versions available online^9^). Next, we synthesize the insights from the breakout sessions held on the final day. Lastly, we share a forward-looking perspective on the future of open data and neurophysiology research.

## II. DEVICES, NEUROINFORMATICS, AND PLATFORMS

*Introductory remarks from the BRAIN Initiative*. In her opening keynote, Andrea Beckel-Mitchener, Deputy Director of the National Institutes of Health (NIH) BRAIN Initiative, commemorated the decennial of the Initiative, a cornerstone in the evolution of brain research through cutting-edge neurotechnologies. She delineated the significant strides made since its inception in 2013, spotlighting the launch of pioneering projects that have enriched our understanding of neural circuits and behaviors across the spectra of health and disease. Beckel-Mitchener lauded the collaborative spirit that has spurred over (US)$3 billion in investments across more than 1300 projects, and synergy among federal agencies, private entities, and the research community. This keynote underscored the symposium’s close alignment with the BRAIN Initiative’s ethos of transparency and the democratization of scientific resources, reflecting a shared ambition for broadening the accessibility and application of data and resources in the scientific and clinical realms.

### A. New Devices and High Throughput Acquisitions

The first session of the symposium on “New devices and high throughput acquisitions” highlighted the latest advancements in neurotechnology, marking a significant shift from traditional methods to innovative approaches that allow for high-resolution, comprehensive recording of brain activity. The session covered both electrical and optical recordings of brain activity at high spatiotemporal resolution. The presenters discussed the development and application of multi-thousand channel electrocorticography grids, volumetric recording at the single-cell resolution across the cortex, advancements in the all-optical electrophysiology study of neuron excitability, and the introduction of Neuropixels NXT for *in vivo* high-density electrophysiology.

These technologies represent a paradigm shift towards more precise methods for studying and treating neurological conditions. They provide insights into the complex dynamics that emerge in neural networks. However, despite the excitement surrounding these advancements, the speakers also highlighted the challenges associated with managing the voluminous data generated, the fidelity of spike sorting, and the importance of recording neurons simultaneously.

The session underscored the critical need for interdisciplinary collaboration, improved computational methods for data handling, and thoughtful consideration of the scientific value versus the practicality of collecting and analyzing massive datasets in neuroscience research. The session concluded with a discussion on the future of high throughput neuroscience, emphasizing the importance of these advancements as well as the challenges that lie ahead.

#### a. Mapping the Human Brain with High Spatiotemporal Resolution.

In the opening talk, Shadi Dayeh (University of California San Diego) discussed the advancements in recording human brain activity using multi-thousand channel electrocorticography (ECoG) grids. He highlighted the shift from traditional clinical electrodes with limited coverage and resolution to modern microelectrode technologies that densely pack thousands of channels into compact areas. This advancement, facilitated by progress in thin-film microfabrication, allows for comprehensive brain activity mapping. Dayeh detailed the technological challenges that needed to be overcome to achieve this, such as scaling down electrode size to increase signal-to-noise ratio and adapting the electrodes to the brain’s curvilinear surface for stable contact^10^. Dayeh also introduced innovative devices like platinum nanorod grids (PtNRGrids) and their clinical applications, from acute to chronic monitoring. He highlighted the move towards wireless systems for efficient and less intrusive monitoring, marking a potential paradigm shift in neurophysiology, both for the experimental and clinical settings.

#### b. High Channel Count Electrophysiology: Present, future.

Neuropixels, a silicon probe which allows high-density simultaneous recording of hundreds of neurons in awake and freely moving animals, have revolutionized systems neuroscience^11^. Tim Harris (HHMI Janelia Research Campus/JHU) discussed the development and applications of Neuropixels NXT, the latest innovation in high-density electrophysiology, emphasizing its capacity to capture neural activity across a broad spectrum of species with unprecedented detail and scale. By integrating multiple components of traditional electrophysiology systems into a single, sophisticated device, Neuropixels NXT represents a significant leap forward in neuroscience research tools, offering researchers the ability to gather data from thousands of neurons simultaneously. Harris highlighted the transformational impact of Neuropixels technology on neuroscience, allowing for more comprehensive and detailed observations of neural dynamics than ever before. However, he also raised critical concerns about the challenges associated with the increased data volume, including issues related to spike sorting fidelity, data management, and the interpretation of vast datasets, questioning the necessity and practicality of recording every neuron simultaneously for meaningful scientific discovery.

Furthermore, Harris reflected on the broader implications of deploying Neuropixels NXT in research, including the potential to change the landscape of primate neuroscience by dramatically increasing the number of neurons observed in a single experiment. He shared insights into the community’s enthusiasm for the technology, as evidenced by the widespread adoption of Neuropixels across various species and research contexts, and the collaborative efforts that made such technological advancements possible. Despite the excitement, Harris expressed reservations about the scientific community’s readiness to handle the deluge of data produced by such high-density recording techniques. He underscored the urgent need for more effective strategies for data compression, sharing, and analysis to fully leverage the technological capabilities of Neuropixels NXT, challenging researchers to think critically about the balance between data collection capabilities and our ability to extract of meaningful insights from complex neural recordings.

#### c. Towards Cortex-Wide Recording of Neuroactivity at Cellular Resolution.

Alipasha Vaziri (Rockefeller University) showcased his lab’s breakthroughs in developing technologies for cortex-wide, volumetric recording of neuronal activity at single-cell resolution, addressing the fundamental question of how sensory inputs and neural activity translate into behavior and computational processes in the brain. His approach, which incorporates light sculpting and temporal multiplexing, expands the volume and scale of neuronal recordings while maintaining the necessary spatial resolution, allowing for the simultaneous recording of activity from millions of neurons across the mouse brain^12^.

This achievement enables unprecedented insight into the complex dynamics of neuronal populations revealing intricate networks of correlated activity across significant distances within the brain. Notably, Vaziri’s findings challenge conventional assumptions about neural data dimensionality. He highlighted evidence that there is a smooth decay in the variance spectrum across thousands of functional brain components. Such an observation, if replicated, would offer a novel vantage point on our understanding of brain function and could inspire future explorations of the anatomical and temporal organization of these higher dimensions of brain activity.

#### d. Voltage Imaging: All-optical electrophysiology of neuron excitability.

Adam Cohen’s (Harvard University) presentation centered on the innovative approach of using voltage imaging to study neuron excitability through an all-optical electrophysiology framework. By leveraging voltage-sensitive fluorescent proteins activated by red light, and blue-light-activated channelrhodopsins for neuronal stimulation, his team has developed a powerful neuro-optical interface. This tool allows for comprehensive monitoring of electrical activity across neuron populations, including spikes and subthreshold voltages, with high spatial and temporal resolution^13^. Cohen’s work aims to unravel the complex dynamics evolving within neurons and their networks by examining the input-output relationships and the plasticity rules that govern changes in neural function.

Again, the vast amount of data generated through these advanced imaging techniques presents significant challenges in terms of analysis, interpretation, and sharing. Cohen pointed out the difficulties in extracting meaningful information from a noisy signal and in distilling the data to manageable proportions for scientific inquiry. Furthermore, he discussed the ongoing struggle to meet NIH mandates for data sharing, emphasizing the need for better tools and methodologies for managing and disseminating large-scale neural imaging datasets. Cohen’s call for improved computational methods to handle these high-dimensional data highlights a critical intersection between neuroscience and data science, suggesting that future advancements in understanding neural dynamics and excitability will likely emerge from collaborative efforts that bridge these fields.

#### e. Concerns & Challenges.

The panel discussion between the speakers and the audience, moderated by Ben Dichter (CatalystNeuro), centered around the utility of collecting extensive neural data, the fidelity of spike sorting, and the practicalities of data compression and sharing. Speakers shared concerns about the reliability of identifying neural units and the potential false positive or false negative spikes derived from current methodologies. It was agreed that signal extraction is challenging, given background noise, the large amount of data to sift through, and the complex relationship between intracellular neuronal excitability and extracellular signatures of neural activity. The discussion highlighted the need for intermediate data compression strategies that ensure data can be feasibly stored, while allowing information essential for reanalysis to be retained. Further discussions underscored that in contrast to imaging, electrophysiological data do not provide precise anatomical insights. It was pointed out that without spatial context, critical information is missing, emphasizing the subjective and artisanal nature of developing models based on such data.

Furthermore, the dialogue explored strategies for efficient data handling, like reducing data dimensionality for more manageable analysis. The Brain Initiative’s concerns about the scalability of online data sharing given the astronomical data generation rates of new technologies prompted a proposal that, instead of sharing the overwhelming volumes of raw data, a more viable approach might involve detailing the methodologies for data acquisition, ensuring others can replicate experiments if needed. This sentiment was also echoed by those advocating for sharing analyzed results and interpretations rather than unmanageable raw datasets. This approach, while it addresses practical constraints, also emphasizes the importance of having experimental and analytical insight into the raw data one is working with. It suggests that a shift towards sharing distilled knowledge and methodologies in the neuroscience community might be more effective.

**Data Management in the Age of High Throughput:** Technological advances, while pushing the frontiers of neuroscience, raise a crucial question for the open science community: how should we manage the terabytes (TBs) of data generated? Large-scale repositories like DANDI are challenged not only by storage needs, but also by the need to efficiently share such voluminous data. Yet, comparisons with data management practices at institutions like the European Organization for Nuclear Research (CERN), which typically handles 50–100 petabytes of data annually^14^, suggest that neurophysiological data repositories likely have the capacity to store and share this data effectively, at least until data generation scales significantly. However, one significant difference between a centralized facility like CERN and the more dispersed laboratories of the neurophysiology community is the ease of access to data engineers possessing the expertise to handle such large throughput. For the former, maintaining a fleet of highly trained personnel is a necessity of operation - for the latter, budgetary concerns can often offload the responsibility to students who lack training in the technical aspects of PB-scale data management. Although comparing current data scales suggests that the problem is not immediate, concerns about storage capacity limits eventually being met prompted a broader dialogue about the principles that should guide a shift towards storing only pre-processed data to ensure the quality and feasibility of data sharing in the long term.

Overall, the discussion highlighted a pivotal moment that may soon be reached in neurophysiology, at which the field’s ability to generate data will outpace its strategies for managing, analyzing, and sharing that data. The conversation pointed towards a need for a paradigm shift in how neuroscientific data is handled, emphasizing the importance of analytical insight and methodological transparency over the indiscriminate sharing of raw data.

### B. Neuroinformatics of Neurophysiology

The neuroinformatics landscape is currently undergoing a transformations, bolstered by significant advancements in data standards, repositories, and computational tools designed to meet the evolving needs of the neuroscience community. These developments are pivotal to addressing the challenges discussed above associated with the management, sharing, and analysis of the rapidly growing volume and complexity of neurophysiology data. This session spotlighted leading innovations in this domain.

#### a. The Neurodata Without Borders Ecosystem for Neurophysiology Data Standardization: Driving collaboration in neuroscience.

Oliver Rübel (Lawrence Berkeley National Lab) focused on the role of Neurodata Without Borders (NWB) as a comprehensive data standard for the neurophysiology community, developed under the NIH BRAIN Initiative with additional support provided by the Kavli foundation. It was emphasized that NWB is not just a singular effort, but a collaborative, multidisciplinary project that incorporates contributions from various researchers and institutions, aiming to address the wide range of needs in neurophysiology data management. This standard facilitates the organization, alignment, and integration of diverse datatypes, from neural activity recordings to experimental metadata by enabling researchers to store all relevant data in a single, hierarchical, accessible format. A highlight of the talk was the detailed overview of the growing NWB ecosystem, which has been expanded to include a range of tools and application programming interfaces (APIs) for data conversion, inspection, and analysis, aiming to lower barriers to adoption and promote widespread use^15^. The talk also addressed the evolving needs of the neuroinformatics community, reflecting the complexity of the analyses deployed on neurophysiology data. Enhancements aiming to address these needs include support for cloud-based data access and analysis, integration with external resources, and efforts to make the standard and its associated tools more accessible to users with diverse skill sets, underscoring NWB’s critical role in the neurodata lifecycle (from acquisition to analysis and sharing) and how it is continuously evolving to meet the community’s changing needs^16^.

#### b. DANDI: An archive and collaboration space for neurophysiology projects.

Satrajit Ghosh’s (Massachusetts Institute of Technology (MIT)) presentation on DANDI (Distributed Archives for Neurophysiology Data Integration)^17^ focused on its role not only as a data repository, but as a collaborative space designed to integrate neurophysiology data across a diverse range of research areas. DANDI, a project supported by the Brain Initiative and AWS public dataset program, and operated in collaboration with MIT, Kitware, and Catalyst Neuro, aims to make neurophysiology data, including but not limited to electrophysiology and optophysiology data, readily accessible and usable for the research community. Ghosh highlighted DANDI’s cloud-based infrastructure, and the fact that it hosts the largest collection of neurophysiology data globally. He also discussed the importance of standards, computing resources, shared ecosystems, and trainings for making data available, discoverable, and usable. He underscored DANDI’s commitment to Creative Commons licensing, its support for standardized data formats like NWB and BIDS, and the ongoing development of tools to facilitate data submission, collaboration, and analysis. Notably, it was emphasized that the vision for DANDI extends beyond data storage to fostering a comprehensive ecosystem for neuroscience research, highlighting the critical need for community-driven standards, curation, and education to enhance data utility and sustainability in the face of rapidly growing data volumes.

#### c. End-to-end Computational Workflows for Neuroscience Research.

Dimitri Yatsenko’s (DataJoint) presentation focused on the development and application of end-to-end computational workflows in neuroscience research, facilitated by tools commercially available from DataJoint^18^. He out-lined the importance of considering a project’s entire lifecycle, from data acquisition to analysis, emphasizing that different stages present unique challenges. He highlighted the diverse needs that arise in neuroscience studies, such as animal management, electrophysiology, spike sorting, and behavior analysis. Through their collaborations with various laboratories, DataJoint has constructed a framework of “operational maturity” in neuroscience research, which assesses a lab’s ability to execute projects efficiently^19^. This model delineates the maturity of systems ranging from ad-hoc processes to AI-enabled operations. The DataJoint Elements resource offers open-source solutions tailored to specific neurophysiology experiments, aiming to elevate laboratories to higher operational maturity levels and foster collaboration within the neuroscience community through standardized workflows and fair data principles^20^. Yatsenko also discussed how laboratories can transition towards more sophisticated levels of operational maturity by, for example, adopting open-source tools and better integrating computational data pipelines. The presentation touched on the future of neuroscience research, depicting AI-enabled workflows as the pinnacle of operational maturity. Examples like the creation of an interactive environment for working with the MICrONS dataset^21^, and the coordination and automation of various collaborative projects illustrate the practical application of DataJoint tools in elevating the operational capabilities of neuroscience laboratories.

#### d. Web-based Visualization and Analysis of Neurophysiology Data.

Jeremy Magland’s (Flatiron Institute) presentation introduced innovative open-source software tools for web-based visualization and analysis of neurophysiology data, highlighting the advantages of leveraging web-based platforms such as ease of use, shareability, and cross-platform functionality. He presented three main tools he has been developing: *Figurl*, a framework for creating and sharing interactive visualizations^22^; *Neurosift*, a tool for browsing NWB files, particularly those hosted on DANDI^23^; and *Dendro*, a prototype web app for analyzing neurophysiology data in the cloud, or using local or cluster compute resources^24^. Magland detailed how these tools facilitate scientific collaboration, reproducibility, and knowledge transfer by simplifying the sharing of interactive figures and visualizations through URLs generated by Python scripts. The integration of these tools with data standards like NWB and platforms like DANDI was emphasized, along with the advantages of client-only applications which do not require server maintenance. Magland’s presentation showcased the potential of these types of tools to revolutionize how neurophysiology data is visualized, analyzed, and shared within the scientific community, generally enhancing the accessibility and collaborative potential of neurophysiology research data.

#### e. Concerns & Challenges.

During the panel discussion, moderated by Yaroslav Halchenko (Dartmouth College), a rich dialogue unfolded on the topic of implementation challenges and ethical considerations that arise when applying neuroinformatics tools and standards to diverse neuroscientific research environments. The key takeaways were:

- *Integration and Utilization of Resources:* In response to an inquiry about how to best integrate and utilize resources in systems neuroscience laboratories, several approaches were recommended, including consulting early with resource developers (such as NWB, DANDI, and DataJoint), integrating standardized processes into workflows to streamline data management and analysis, and publishing effective workflows to share them with the community.
- *Usability:* Relatedly, the panel discussion touched on the critical need for enhancing the usability of neuroinformatics tools and ensuring robust user support to facilitate widespread adoption. Panelists concurred that maintaining a low barrier to entry is essential for enabling researchers to effectively integrate these sophisticated tools into their workflows, thereby ensuring the community can keep pace with the rapidly increasing volume and complexity of neurophysiology data.
- *Standardization and Risk Management:* The panelists pointed out the necessity of standardizing data acquisition systems to facilitate data sharing and analysis. This standardization can also help address risks associated with data identifiability to ensure that shared data complies with privacy standards (in clinical settings).
- *Transparency and Reproducibility in Cloud-based Analysis:* Panelists argued that automated workflows and containerization technologies enhance the reproducibility and observability of computational research, making cloud-based analysis a viable and transparent option for neuroscientific research.

Overall, the discussion underscored both the difficulties and opportunities that arise when adopting neuroinformatics tools and standards, highlighting the importance of community collaboration, ethical considerations in data sharing, and the potential benefits of cloud-based computational workflows for the future of neuroscience research.

In summary, NWB has emerged as a comprehensive data standard, fostering a collaborative, multidisciplinary effort to streamline neurophysiology data management, and the creation of intuitive tools such as NeuroConv and NWB GUIDE has helped simplify the conversion of proprietary data into the NWB format. These initiatives reflect a concerted effort to enhance the accessibility and usability of data sharing between research laboratories, and position NWB as a cornerstone of neurophysiology data management. Reflecting its rapid growth, the DANDI repository currently hosts an impressive 276 TB of public neurophysiology data and is poised to play a pivotal role in shifting the neuroscience community toward open science. DANDI’s rapid expansion is a testament to its inclusive approach, accommodating a wide spectrum of neurophysiology data, from raw to processed forms, across various species. This versatility broadens the repository’s utility and sets a leading example for the burgeoning ecosystem of tools and standards facilitating open science practices.

Further enriching this landscape are emerging commercial services and web-based tools that aid laboratories in scaling their data management and analysis capabilities. Services such as DataJoint and CatalystNeuro offer tailored software solutions, enhancing operational efficiency and supporting end-to-end data lifecycle management. Innovative web-based platforms like Neurosift, Dendro and Figurl represent technological advancements supporting accessible, collaborative neuroscience research. These tools enable seamless interaction with complex datasets and foster scientific collaboration through shared, interactive visualizations, highlighting a shift towards more accessible and collaborative neuroscientific research.

Integrating these standards, repositories, and tools reflects a collective stride towards addressing the neuroinformatics community’s growing needs, and underscores a pivotal moment in the evolution of neurophysiology research. As we navigate this era of transformation, the continual development and adoption of these resources will be instrumental in enhancing data utility, promoting open science, and advancing our understanding of the brain.

### C. Platforms/Infrastructures

This third session focused on the critical importance of collaboration, standardization, and open science in advancing our understanding of neurophysiology and tackling the reproducibility crisis in neuroscience research. A shared goal emerged across the talks for creating integrated databases and analytical frameworks that not only facilitate the exploration of neuronal activity and brain function across species, but also provide invaluable resources for the global scientific community. Efforts in this direction underscore an ongoing transition towards inclusive, transparent, and collaborative research infrastructures, promising to accelerate discoveries in neuroscience. The session highlighted not only the advancements in neuroscientific research methodologies and technologies, but also the challenges ahead in achieving consensus on data interpretation, and the need for continued innovation in data analysis and sharing practices.

#### a. Brain Mapping and Disease Modellings using Genetically Modified Marmosets.

Hideyuki Okano (Keio University/Riken) laid out the Japan’s Brain/MINDS project’s groundbreaking work in brain mapping and disease modeling using genetically modified marmosets. The initiative has made significant contributions to open science by publicly sharing the collected marmoset datasets, including structural, diffusion and resting-state MRI datasets, as well as quantitative 3D data, and an *in situ* hybridization-based marmoset gene atlas^25–27^. By integrating gene expression and brain structure data, this comprehensive database serving as a valuable reference for detecting abnormalities in disease models and facilitating interspecies comparisons. The initiative’s research has notably revealed, among other discoveries, between and within-column connectivity patterns in the prefrontal cortex of marmosets that are not observed in mice. The initiative also developed models of certain neurodegenerative and neurodevelopmental diseases. This has enabled, for example, a detailed study of Rett syndrome using CRISPR Cas9, which revealed reduced connectivity, poor dendritic arborization, and a disruption in excitatory/inhibitory balance due to hypermaturation of parvalbumin neurons. These studies also showed that MECP2 knock-out marmosets display gene expression changes similar to those observed in human patients, bolstering the potential of this research to provide insights into the molecular mechanisms underlying Rett syndrome, and to reveal potential therapeutic targets.

#### b. OpenScope: The first astronomical observatory in neuroscience.

Jérôme Lecoq (Allen Institute for Neural Dynamics) gave an introduction to the Brain Observatory, a vast database of cellular-level activity in the mouse visual system, and to OpenScope^28^, a platform allowing external scientists to propose a project for which high-throughput and reproducible neurophysiology data is then collected. He highlighted these projects as pioneering initiatives in the community, poised to transform the landscape of neuroscience research, and emphasized their significance as being akin to having an astronomical observatory for neurophysiology^29,30^. Lecoq also underscored the collaborative nature of these projects, spotlighting the essential role of a wide range of professionals, from scientists and engineers to animal care staff, in their success. He traced the evolution of neurophysiological research methodologies over the past decade, showcasing the development of pipelines for two-photon microscopy and Neuropixels recordings in the Brain Observatory, and of OpenScope platforms that have facilitated comprehensive analysis of brain activity in behaving mice. Lecoq demonstrated the impact of these efforts through the growing number of scientific publications exploiting these datasets and discussed the operational model of OpenScope, which allows researchers to submit projects for consideration in a manner analogous to time allocation for shared telescopes in astronomy.

The core of Lecoq’s talk focused on the OpenScope project’s operational model and future aspirations. OpenScope permits researchers globally to submit project proposals through a process that aims to be highly equitable and inclusive of researchers who would not otherwise have access to such resources. As such, this model not only maximizes research efficiency and community engagement, but also fosters scientific innovation by breaking down traditional barriers to data access and tool utilization. Lecoq detailed the rigorous, double-blinded review process designed to minimize bias and ensure that projects are selected based on scientific merit. He also discussed the platform’s ongoing efforts to enhance the neuroscience data analysis ecosystem, such as the development of the OpenScope Databook^31^. This initiative aims to democratize access to expertise in sophisticated data acquistion techniques and support the community’s increasing use of standardized computational tools. Lecoq’s insights into the challenges involved in updating the community’s technological toolkit, coupled with his recognition of the substantial financial investment required to run these high-caliber projects, illustrate the pivotal role platforms like OpenScope may play in shaping the future of neuroscience research.

#### c. Compute, Data & Standards in Large-Scale Neuroscience.

David Feng’s (Allen Institute for Neural Dynamics) talk on open data in neurophysiology delved into the crucial roles of computing, data management, and standards within the context of large-scale neuroscience research, specifically at the Allen Institute for Neural Dynamics. Feng introduced the Institute’s ambitious mission to uncover the neural underpinnings of emotions, memories, and actions, utilizing advanced neurotechnology tools to simultaneously capture comprehensive brain-wide recordings and extensive behavioral data.

He emphasized the Institute’s commitment to open science, highlighting their efforts to make vast amounts of data widely accessible and useful to the scientific community. Feng discussed the challenges associated with managing, sharing, and analyzing petabytes of data, reinforcing the need for robust, human- and machine-readable metadata generated at the time of data acquisition and moving beyond the common practice of creating retrospective documentation at the time of publication. This approach supports making data FAIR (Findable, Accessible, Interoperable, Reusable) immediately upon collection, by leveraging community standards like BIDS, NWB, and OME.

Additionally, Feng pointed out the importance of cloud computing for enhancing the utility and inclusiveness of open science initiatives. He elaborated on the following advantages of cloud platforms in:

- Reducing the logistical challenges associated with moving and storing terabytes to petabytes of data.
- Simplifying the sharing of complete software and hardware environments, making reproducible science feasible on a large scale with tools such as *“Code Ocean”* and *“GitHub Codespaces”*.

He also addressed the critical need for fully reproducible processing pipelines in neuroscience. Despite the existence of many relevant tools, most present installation difficulties, show inconsistent performance across different environments, and involve a myriad of lightly documented parameters that require fine-tuning. To overcome these hurdles, the Allen Institute is developing containerized *“NextFlow”* pipelines tailored to neurophysiology data. These pipelines are designed to operate efficiently in both cloud environments and on-premise infrastructure, ensuring they are accessible and beneficial to the broader neuroscience community.

This vision for the Allen Institute mirrors a broader shift towards transparency and accessibility in neuroscience, and aims to foster a more collaborative and efficient research ecosystem.

#### d. International Brain Laboratory: A brain-wide map of neuronal activity during behavior.

Matteo Carandini (University College London) introduced the groundbreaking work of the International Brain Laboratory (IBL), a collaborative effort involving 22 laboratories across various countries to create a brain-wide map of neuronal activity during behavior in mice^32^. Carandini discussed common challenges faced in neuroscience research, such as reproducibility problems and discrepancies in experimental findings across different studies. He highlighted the IBL’s mission to overcome these challenges by jointly developing standardized experimental protocols and then pooling the data collected by participating laboratories. The project aimed to understand how brain-wide circuits underpin complex behaviors, exploiting in particular the ability of Neuropixels probes to capture neuronal activity across many brain areas simultaneously during a behavioral task. The IBL’s approach yielded a massive dataset from nearly 33,000 neurons, enabling a comprehensive analysis of how different brain regions process information related to sensation, decisions, actions, and prior beliefs^33–35^.

Carandini also touched on challenges encountered by the IBL. In particular, despite achieving significant reproducibility and uncovering consistent and widespread encoding of behavioral information across the brain, the IBL team found that different analytical methods used on the same data could nonetheless yield distinct interpretations. This problem underscores a deeper issue in neuroscience: the difficulty in achieving consensus on interpretations and conclusions drawn from neuroscientific data. The IBL’s approach, focusing on reproducibility and standardization across an international collaborative network, argues for the value of developing unified methodologies and emphasizing open science practices to address these challenges. The variability in analytical outcomes, nonetheless, serves as a reminder that our grasp on the tools themselves that we use to understand the brain remains limited, and is jostled by each new discovery and methodological advancement. Thus, we must continue to exercise caution in drawing conclusions about the intricacies of brain function.

#### e. Concerns & Challenges.

The panel discussion following the session was moderated by Katherine Fairchild (MIT). It delved into several critical topics related to the collection, sharing, and reuse of neuroscientific data. The crucial role of metadata and auxiliary data in enhancing the value and applicability of neuroscience datasets was emphasized, and several broader insights about the advancement of neurophysiology research and the effective use of open data platforms were raised:

- *Importance of Comprehensive Metadata:* There was a consensus on the necessity of capturing extensive metadata to account for as many variables as possible that might affect experimental outcomes. This includes environmental conditions, experimental protocols, and even minor details that could influence the data, such as the presence of specific individuals during the experiments^36^.
- *Challenges in Metadata Collection:* It was also discussed how difficult is can be to accurately capture and maintain metadata, and that there is a lack of assistive tools that are both comprehensive and user-friendly. This makes it challenging to consistently record essential data, which is crucial for replicating and understanding the context of experiments.
- *Data Privacy and Transparency:* The conversation touched on the need to balance data sharing with privacy, particularly when recording potentially sensitive information within experimental settings. In particular, the discussion underscored the complexity of managing open data while respecting privacy and confidentiality.
- *The Need for Hypothesis-driven Research:* There was a call for more hypothesis-driven approaches when collecting and sharing large-scale data. This includes the development of benchmarking platforms that would allow researchers to test specific predictions and hypotheses, potentially addressing the challenge of data over-collection and focusing research efforts on the comparative testing of theoretical models.
- *Engaging Data Analysts:* The panel identified a disconnect in the neuroscientific community where the efforts and concerns of data analysts, those who primarily analyze and derive insights from existing datasets, are not well integrated in the modes of operation of experimental laboratories. Engaging this group more effectively could provide valuable feedback on data and metadata needs, potentially guiding more efficient and targeted data collection and sharing practices.
- *Diversity of Research Approaches:* The discussion acknowledged the diversity of approaches in neuroscience, from data-driven to hypothesis-driven research, reflecting the broad range of questions and methodologies represented in the field. This diversity gives rise to a need for flexible and adaptable data platforms that can accommodate different research needs and objectives.
- *Future Directions and Community Engagement:* The conversation emphasized the importance of community engagement in developing and refining data sharing platforms. This includes not just tool developers, but also those focused on using shared data for discovery, suggesting a collaborative approach is needed to improve data usefulness and accessibility for the neuroscientific community at large.

The Japan Marmoset Initiative, the International Brain Laboratory, and the Allen Institute for Neural Dynamics exemplify a shift towards large-scale, collaborative neuroscientific projects. These initiatives not only harness cutting-edge technologies for brain mapping and disease modeling, but have also pioneered the development of comprehensive, integrated databases accessible to the global scientific community. Such collaborative projects, which transcend traditional single-laboratory models, are crucial to tackling the operational and analytical challenges posed by the scale and complexity of contemporary neurophysiology research.

These initiatives also underscore the critical importance of open, reproducible research in neurophysiology. Their commitment to sharing large-scale neural circuit mapping data, accompanied by public analysis pipelines exemplifies a forward-thinking approach to scientific inquiry. Importantly, it enables researchers worldwide to engage with complex datasets without extensive software engineering expertise, fostering a more inclusive and collaborative scientific community.

The discussions surrounding the sustainability of storing and sharing vast amounts of raw data, alongside the complexity of capturing standardized metadata, highlight critical infrastructural challenges for neurophysiology research. As neuroscience research evolves, the community should continue to explore balanced strategies for data lifecycle management, emphasizing the selective maintenance of high-reuse datasets and the development of automated metadata capture systems.

## III. KNOWLEDGE EXTRACTION, SOFTWARE, MODELING

### A. OpenData2Knowledge

This session was a deep dive into how the open sharing of neuroscience data facilitates scientific discovery, enabling researchers to build upon each other’s work and accelerate the pace of innovation. The speakers presented a range of approaches, from the use of organoids and data-driven models to applications of computational techniques and innovative recording technologies, highlighting the interdisciplinary effort required to decode a complex system like the brain. The overarching theme of the session was the exploration of novel methodologies and technologies to address longstanding questions in neuroscience, such as the mechanisms underlying neural circuit development, the processing of complex sensory information, and the generalization capabilities of the brain compared to AI systems.

#### a. Intrinsic Activity In Human Cortical Organoids Reveal Protosequences that Model Default States in the Developing Cortex.

In his talk, Kenneth Kosik (University of California, Santa Barbara) delved into how organoids can be harnessed to study network development and electrical signaling within neural circuits, specifically through the use of integrated optofluidic-CMOS multielectrode arrays^37^. He opened his presentation by addressing skepticism around organoids, arguing that their value should not be judged solely based on their similarity to the brain, but also on how they have propelled the field beyond two-dimensional neuronal cultures. This transition has produced significant new biological insights, including discoveries related to the lamination patterns and local field potentials which emerge in organoids, but are not observed in dissociated cultures.

Kosik emphasized the usefulness of organoids for modeling the intrinsic activity of the cortex, in the absence of experiential input, using advanced technologies like MaxWell Biosystems microelectrode arrays and Neuropixels to explore network dynamics and the distribution of various cell types and neurotransmitter receptors. Further, Kosik shared the potential of organoids for modeling learning processes, while acknowledging the speculative nature of such applications. He discussed both the possibilities for inducing learning and memory formation within organoids, and related challenges, describing experiments in which repeated stimulation is used to mimic repeated sensory input. These endeavors illustrate the evolving interplay between neuroscience and technology, with the development of organoid research potentially opening a new door for investigating in a versatile way neural development, network dynamics, and potentially, the mechanisms underlying learning and memory.

#### b. Data-driven Dynamic Models of Large-scale Neural Data.

In her presentation, Bing Brunton (University of Washington) explored the potential of data-driven models to enable researchers to decode and understand large-scale neural data. She described a project in which video-annotated human electrocorticography recordings were used to study, in a natural setting, the relationship between brain activity, and complex and dynamic natural behaviors, with the aim of improving brain-computer interface technologies^38^. This research not only advances our understanding of brain activity in realistic contexts, it also exemplifies the transformative power of open science and collaborative research in advancing the clinical applications of neurophysiology research. By making their findings and methodologies publicly available^39^, Brunton’s laboratory enabled new discoveries to be made by other researchers, supporting inclusion and collaboration within the scientific community. The AJILE12 dataset has also been integrated into Neuromatch Academy Projects, providing students around the world with firsthand experience analyzing real data^40^.

#### c. A Less Artificial Intelligence: Exploring mechanisms through MICrONS.

Andreas Tolias (Stanford University) presented an insightful exploration of cortical networks through the lens of the MICrONS project, tackling the enduring question of what distinguishes the cognitive capabilities of the brain from the capabilities of AI systems. The MICrONS project, a collaborative scientific endeavor, provides an open and publicly accessible data portal for accessing connectivity and functional imaging data collected by a consortium of laboratories. These data include large-scale electron microscopy-based reconstructions of cortical circuitry from mouse visual cortex, along with corresponding functional imaging data from some of those same neurons^41^.

Tolias highlighted the brain’s exceptional ability to generalize from limited datasets—a feat AI continues to struggle with. He explained how projects like MICrONS have the potential to revolutionize machine learning by guiding us in reverse-engineering the brain’s algorithms. By employing innovative methodologies like “inception loops”, whereby a neural network is trained on neurophysiological recordings to discover stimuli that maximally excite specific neurons^42^, his team was able to uncover previously unappreciated organization features of visual processing circuits. This approach provides grounds for comparing biological and AI performance, but more importantly charts a course for integrating biological insights into the development of more sophisticated AI systems. Tolias’ exploration of the cortical structure and function, as part of the MICrONS project, sets a precedent for future multi-team research projects aimed at deciphering the neural code underlying the brain’s unique cognitive abilities. In addition, by making this data available to researchers worldwide, MICrONS fosters transparency, accelerates scientific progress, and helps bridge the gap between biological insights and the development of sophisticated AI systems.

#### d. The Role of Inhibitory Neurons in Auditory Processing.

Maria Geffen’s (University of Pennsylvania) presentation delved into the intricate roles of inhibitory neurons in the auditory cortex, focusing on how these cells influence phenomena related to auditory processing and perception, like frequency discrimination and adaptation to temporal regularities in sound. Her laboratory uses a combination of opto-electric recordings and computational techniques to discern the influence of different types of inhibitory neurons on auditory perception and network dynamics, particularly in the context of complex sound processing^43,44^. Through this approach, she has demonstrated that manipulating specific inhibitory neurons can profoundly affect the brain’s ability to discern frequencies, thereby impacting auditory perception at a fundamental level.

#### e. Concerns & Challenges.

The panel discussions, moderated by Colleen Gillon (Imperial College London), opened with reflections on challenges and successes in making neurophysiological data accessible and useful for broad scientific endeavors. Highlighting the importance of platforms like GitHub and of data repositories, speakers discussed their strategies for ensuring their data and code are not just perfunctorily made available, but also genuinely reusable by the community. This part of the discussion highlighted a recurring theme from the symposium: the pivotal role of effective data sharing and the necessity of including comprehensive metadata to enhance data utility for different research groups. The key takeaways for how to steer the community towards novel discoveries and theoretical advancements were:

- *Open Science Practices:* Panelists highlighted the importance of sharing data and code through common platforms, underscoring a commitment to open science principles that facilitate wider accessibility and reuse of research outputs.
- *Challenges in Data Reuse:* A significant challenge discussed was navigating the plethora of data repositories available and identifying the best sources for specific data. This concern reflects the need for better guidance on where to find reusable data, as well as the importance of providing comprehensive and searchable metadata to ensure datasets are findable, understandable and usable without direct communication with the original authors.
- *Importance of Metadata:* The panel re-emphasized that well-documented metadata is crucial for effective reuse of datasets. Metadata quality directly impacts the ability to understand an existing dataset and apply it to new research questions without needing to consult the data creators.
- *Integration of Dynamical Systems Theory:* The panelists strongly advocated for applying dynamical systems theory to neuroscience. This reflects the shared view that this mathematical lens is best suited to exploring the temporal patterns that emerge in biological systems.
- *Need for New Mathematical and Computational Models that Can Meet Challenges in Modeling Complex Systems:* During the discussion, participants highlighted the limited ability of current mathematical frameworks to fully capture the complexity of neural systems. There was a consensus on the necessity of developing new computational methods that can provide more flexible and stable solutions for modeling biological data. Additionally, the difficulties inherent in modeling nonlinearity, non-stationarity, and the sheer scale of neural data were emphasized. These challenges will necessitate the development of novel computational tools and mathematical approaches capable of handling this level of complexity.
- *Digital Twins and Intuitive Modeling:* The concept of “digital twins” (a virtual representation of an object or system designed to accurately reflect the dynamics and processes of that object or system) and the idea of incorporating scientific intuition and expertise into machine learning models were discussed as promising directions for future research. These approaches offer one way to meaningfully incorporate the insights gained from decades of scientific exploration into detailed system models.
- *Perspectives on Understanding through Analytical vs. Numerical Methods:* There was a debate on the value of analytical solvability versus numerical approaches for gaining true conceptual insight into systems. While some panelists and audience members argued that analytical methods are indispensable for gaining deep understanding of a system, others advocated for the pragmatic use of numerical methods and data-driven approaches to tackle complex systems like the brain.
- *Future Directions in Neuroscience Research:* The panel discussion concluded with a forward-looking perspective on neuroscience, emphasizing the importance of integrating knowledge across disciplines and methodologies. This means embracing dynamical systems, control theory, and developing novel mathematical frameworks to better understand and model the brain’s dynamic behaviors and its interactions with the physical world.

### B. Neuroscience Toolkits

A common thread running through the presentations in this session was the innovative use of digital tools and machine learning to tackle complex questions using neurophysiological data, breaking new ground in how we study and understand neural mechanisms. From the nuanced dissection of animal behaviors to the standardization of electrophysiological data analysis, and from harnessing machine learning to decode neural activity patterns to leveraging the vast collaborative networks of large projects, each talk highlighted the importance of promoting synergy between technology and neuroscience. This convergence in ideas reflects not only significant advances in individual tools or methodologies, but also a broader movement towards democratizing science, enhancing access to these tools, and fostering a global scientific community united by the shared goal. Table I provides a summary of the neuroscience toolkits presented in this symposium.

#### a. Linking Large-scale Neural Data to Behavior: Algorithms & opportunities.

In her keynote talk, Mackenzie Mathis (École Polytechnique Fédérale de Lausanne) emphasized the importance of approaching neurophysiology and animal behavior data through a shared scientific lens. She demonstrated the pivotal role cutting-edge computational algorithms and AI play in enabling researchers to decipher the relationship between neural activity and complex behaviors. For example, “DeepLabCut”^45^, an AI-based toolkit developed by her laboratory, has revolutionized the quality and efficiency of automated pose-estimation from animal recordings^46^. Mathis also introduced “Cebra,”^47^, an encoding tool for learning for joint embeddings of behavioral and neural data^48^. These tools exemplify the power of open science and the practical applications of AI for quantifying and understanding behavior. Importantly, these contributions extend beyond the development of analytical tools to an active investment by and for the community in their long-term adoption, reliability and longevity. Mathis’ work presents a clear example of how scientific inquiry is democratized when advanced computational resources are made openly available to the broader scientific community.

#### b. MoSeq (Motion Sequencing): Quantifying 3D video of freely behaving animals.

Bob Datta (Harvard Medical School) introduced MoSeq (Motion Sequencing), a cutting-edge tool for parsing 3D videos of freely behaving animals, emphasizing how it can be used to codify the complex and dynamic behaviors of animals in naturalistic settings. MoSeq utilizes depth cameras to capture detailed 3D movements of rodents, provides insights into their natural behaviors by identifying distinct behavioral “syllables”, and constructs behavioral state maps to help researchers understand the sequential and contextual structure underpinning these behaviors^49^. This unsupervised machine learning approach not only dissects the intricate patterns underlying animal behavior, but also allows researchers to explore the impact of various external perturbations on these behavioral patterns. Datta highlighted MoSeq’s potential contributions to neuroscience research, amongst other things through its ability to distinguish between the effects of different drugs on behavior and reveal variability in behavior across individuals that is both significant and consistent over time. The talk also delved into the technological advancements and collaborations that have enhanced MoSeq’s applicability, such as the integration of key-point tracking to improve data quality in complex environments. With the open and accessible nature of this tool, and its robustness and versatility, MoSeq represents another significant contribution to the field, poised to gain wide adoption and continue to develop through collaborative contributions^50^.

#### c. SpikeInterface: Spike sorting in large-scale recordings.

Alessio Buccino (Allen Institute for Neural Dynamics) highlighted developments and advancements in SpikeInterface, a comprehensive Python package designed to simplify and standardize the spike sorting step in electrophysiology data processing^51^. Buccino detailed the challenges faced in the field, such as the wide variety of acquisition systems and of file formats, along with the lack of reproducibility across toolboxes and often missing data provenance information. SpikeInterface addresses these by providing a unified interface for comparing the outputs of various spike sorting algorithms and pre-processing tools, applying them to data and generating detailed pre-processing reports. The initiative, which began in 2018 through a collaboration across multiple institutions, aims to tackle the fragmentation in electrophysiology data analysis by offering an easy-to-use, standardized solution. SpikeInterface supports over 15 spike sorters and facilitates the entire spike sorting pipeline, from pre-processing and sorting to post-processing and visualization, all while enabling reproducibility and community development^52^.

In the latter part of his talk, Buccino described recent features added to SpikeInterface to enhance its compatibility with cloud-based processing, showcasing its efficiency in handling large-scale recordings. These advancements include a data compression framework that significantly reduces the file size of recordings^53^, streamlined and reproducible pipelines that accommodate various computational backends, and web-based, shareable visualizations for quality control and manual curation. These features not only make SpikeInterface a more versatile tool for researchers, but also foster a community-driven approach to improving electrophysiology data analysis. Going forward, community engagement, including active feedback, will be critical not only for refining tools like SpikeInterface, but also, more broadly, addressing evolving challenges in electrophysiology research.

#### d. Machine Learning Tools for Understanding Complex Hippocampal Patterns in Learning and Memory.

Andrea Navas-Olive (IST-Austria) presented new machine learning tools for analyzing electrophysiological data, with a specific focus on sharp wave-ripples (SWRs), which are crucially involved in memory consolidation. She discussed challenges in detecting SWRs due to the variability in their statistics, and how traditional spectral methods might bias the types of events that are detected. Employing machine learning techniques, she and her colleagues have developed algorithms that improve detection performance, while reducing dependency on often arbitrarily selected thresholds which can bias the characteristics of detected events^54^. These algorithms are not only applicable across different areas of the brain but also generalize to other species, demonstrating their potential for broad applications in neuroscience research^55^. The innovative aspects of this work extend beyond the development of sophisticated detection algorithms; they also encompass the collaborative, crowd-sourced approach to problem-solving behind the project. Navas-Olive co-organized the BrainCode Games hackathon, engaging a diverse group of participants from various backgrounds to tackle the challenge of SWR detection. This collaborative effort not only led to the creation of multiple effective machine learning models, but also fostered the development of a community of interdisciplinary researchers and industry professionals united by a common goal. The hackathon’s success highlights the value of inclusive, community-driven research endeavors for generating unbiased, comprehensive solutions to shared problems. Thus, in addition to producing significant scientific findings, this work also showcased a novel approach for engaging a broad spectrum of talents to advance neuroscience research^56^.

#### e. Neural Ensemble & HBP/EBRAINS Knowledge Graph.

Andrew Davison (Université Paris-Saclay/Centre National de la Recherche Scientifique) focused on the achievements of the Human Brain Project (HBP) and its integration in the EBRAINS Knowledge Graph^57^, two initiatives aimed at advancing digital neuroscience. The HBP, a decade-long EU-funded project with a budget of approximately 600 million Euros brought together over 500 researchers and 100 universities to push the boundaries of science and engineering in pursuit of understanding the human brain. A culminating outcome of this project was the establishment of EBRAINS, a comprehensive research infrastructure offering digital tools and services for neuroscientists. Davison highlighted the transformative potential of EBRAINS for facilitating data-driven science, emphasizing its role in providing sustainable, high-quality digital resources for the neuroscience community. The EBRAINS Knowledge Graph, a core component of this infrastructure, serves as a universal metadata repository for the project, enhancing data discoverability and interoperability. This digital repository enables the sharing of experimental data, computational models, and software tools, all interconnected within a semantic, linked data framework^58,59^. Through its sophisticated architecture and community-driven approach, the EBRAINS initiative exemplifies the importance of collaborative science and open data when tackling the most complex questions in neuroscience.

#### f. Concerns & Challenges.

The panel discussion following these talks, moderated by Ryan Ly (Lawrence Berkeley Lab), centered on critical challenges that emerge when developing, maintaining, and sustaining open-source tools for neurophysiology research. Panelists shared their personal experiences and the various hurdles they’ve encountered, such as transitioning project responsibilities, securing funding, and fostering community engagement. A recurring theme was the essential role of financial support in not only developing these tools, but also in hiring dedicated personnel to maintain and promote these resources, and support potential users. Despite these challenges, there was an underlying optimism about the future, with mentions of the field’s gradually improving recognition of the importance of funding open-source software development. The key takeaways covered a variety of topics, including:

- *Transitioning Project Responsibilities:* A challenge highlighted was how project maintenance is disrupted and can fail entirely when the original developers, often PhD students or postdocs, leave the laboratory. This constitutes a major barrier to the longevity and reliability of open-source tools.
- *Funding for Maintenance:* Panelists unanimously agreed that securing funding is a major hurdle for the development and maintenance of open-source tools. Financial support is necessary not only for initial development, but also for ongoing maintenance, updates, and community engagement efforts.
- *Importance of Dedicated Personnel:* Relatedly, the importance of investing in dedicated personnel, such as community managers and full-time developers, was emphasized. Roles like these are critical for promoting a tool, supporting users, and ensuring the tool remains up-to-date, reliable, and user-friendly.
- *Community Engagement and Collaboration:* Engaging the wider community and fostering collaboration were highlighted as key to the success and sustainability of open-source projects. Contributions can range from fixing documentation to adding new features, enhancing a tool’s quality and applicability.
- *Automation and Testing:* Implementing automation, continuous integration, and comprehensive testing protocols is essential for ensuring the reliability and stability of open-source tools, facilitating maintenance and updates.
- *The Role of Code Readability:* Ensuring that code is readable and standardized is important for making open-source projects accessible to new contributors, and makes it easier for users to understand and use software.
- *Structural Changes in Research Support:* Panelists called for structural changes in how research and development are funded and supported, advocating for a model that recognizes and funds software development as an integral part of scientific research.
- *Increased Recognition of Software Development in Academia:* Relatedly, it was recognized that there is a promising growth in acknowledgment within the scientific community and among funding bodies of the importance of supporting open-source software development. This is seen as a positive trend towards improving the sustainability of digital tools in research.
- *Challenges to Reproducibility with AI Classifiers:* The discussion touched upon challenges to ensuring reproducibility when using AI classifiers, emphasizing the need for classifiers to be re-generated and validated as part of the research process to ensure reliability.
- *Annual Hackathons and Community Events:* The idea of organizing annual hackathons or community events was proposed as a strategy to maintain momentum, engage a broader audience, and generate fresh ideas for the continued development of open-source tools.

### C. Modeling and Benchmarking

The last session covered a wide range of topics, from benchmarking frameworks and integrative modeling to investigating neural variability and dynamics underlying learning and memory. A common theme that emerged from these talks was the importance of going beyond simple analyses performed on isolated experiments in neuroscience, toward a more holistic approach combining sophisticated computational tools and models, with large-scale collaborative data collection efforts. This shared viewpoint highlights a pivotal shift towards leveraging computational neuroscience not just as a tool but as a foundational pillar for elucidating the intricacies of brain function, behavior variability, and the underlying neural representations.

#### a. Brain-Score (Integrative Benchmarks For Models at Scale).

Martin Schrimpf’s (École Polytechnique Fédérale de Lausanne) talk shed light on the importance of harnessing large-scale datasets and establishing experimental benchmarks for advancing brain modeling techniques. Schrimpf emphasized the collaborative effort required in this burgeoning field, noting that the accumulation of large, but disconnected, datasets, while valuable, is not sufficient by itself for comprehensive brain modeling. He argued that disparate datasets should be connected through approaches like integrative benchmarking, which streamline how data are used to evaluate models and compare them to one another. This, in turn, provides more unified and effective guidance and constraints for the development of new and improved models^60–62^. This strategy is exemplified by the Brain-Score platform^63,64^, a tool designed to evaluate models on a wide array of neural and behavioral tasks, offering a holistic approach to identifying the class of models that best recapitulate the brain’s functions^65^. Schrimpf also demonstrated how models of the brain can be used to predict experimental outcomes and optimize data collection, suggesting a future where such models can crucially inform experiment design and neural data interpretation.

#### b. Taming Machine Learning Models of Neural Dynamics with Anatomical and Behavioral Constraints.

Shreya Saxena (Yale University) delved into the complexities of the neural computations underlying motor functions. She emphasized the importance of integrating biophysical and anatomical constraints into machine learning models, demonstrating how this improves the ability of models to recapitulate the neural activity observed during movement and interactions between subjects. One aspect of her research involves dissecting the neural encoding strategies that underlie flexible motor control, i.e., how the motor cortex adapts its activity patterns to drive different muscle movements as tasks change. Considering how different variables, such as visual signals and individual muscle movements, contribute to coordinated actions, Saxena proposed a novel approach that combines traditional neural network models with detailed biophysical knowledge. In this approach, changes in motor task demands are analyzed to determine how they affect neural representations in the motor cortex. These representation changes are then studied to understand how they guide the activation patterns of different muscle groups that allow animals to make precise adjustments to their movements^66–68^. This approach aims to enhance the ability of models to generalize across different conditions and tasks by grounding them in relevant physiological and anatomical constraints. In addition to directly improving models of the neural basis of motor control, this approach has the potential to generate computational models that could contribute to shared databases and frameworks.

#### c. Low-dimensional Manifolds for Neural Population Dynamics.

Hannah Wirtshafter (Northwestern University) offered an insightful exploration of the dynamic learning and memory representations in the hippocampus, particularly focusing on how they evolve based on spatial context. Wirtshafter discussed a specific hippocampal-dependent task, Trace Eyeblink Conditioning, that is used to examine how animals generalize learned responses across different environmental contexts. Using calcium imaging and open-source analysis tools, she observed that animals were able to rapidly reapply conditioned responses when transitioning between environments, despite extensive place cell remapping in their hippocampus. This adaptability raises intriguing questions about the neural mechanisms that maintain task-specific learning against the backdrop of changing spatial representations. Through preliminary analyses using a variety of dimensionality reduction and data analysis techniques, Wirtshafter aimed to uncover whether the neural representations of space and of the task were maintained within distinct or overlapping neural manifolds, in order to gain insight into the complex interplay between spatial navigation and memory processes in the hippocampus. Wirtshafter’s work comparing insights drawn from different data analysis techniques, and in particular different dimensionality reduction techniques, highlights the importance of shared libraries documenting the applicability and theoretical grounding of different analytical tools and methodologies used in neuroscience research.

#### d. Quantifying Animal-to-animal Variability in Large-Scale Neural Recordings through Shape Metrics.

Alex Williams (Flatiron Institute/NYU) highlighted both the challenge and potential value of quantifying animal-to-animal variability in large-scale neural recordings for advancing systems neuroscience research. Emphasizing that comparative approaches are a fundamental methodology in biology for understanding complex systems, Williams argued that such methodologies are underutilized in systems neuroscience, largely due to limitations in experimental techniques and the lack of scaled collaborative efforts. Leveraging data from initiatives like the IBL, in which standardized data was collected across multiple laboratories, Williams demonstrated how “shape metrics” can be used to compare neural representations or manifolds across different animals in a high-dimensional space, irrespective of individual differences in neuron populations^69–72^. Using this approach, which draws on principles from shape theory, he aims to develop open-source tools for analyzing neural data that go beyond traditional R-squared scores, and instead allow models to be matched to specific hypotheses. Williams’ work leveraging IBL data shows how understanding the neural basis of behavioral variability can help us elucidate the links between distinct neural representation patterns in brains and the diversity of learning and performance outcomes observed across individuals.

#### e. Concerns & Challenges.

The following panel discussion, moderated by Manuel Schottdorf (Princeton University), highlighted several critical concerns for current neuroscientific research^8^, and emphasized the importance of benchmarks, interpretability, and the quality of input data. A consensus emerged on the necessity of using both supervised and unsupervised methods in neuroscience research, with benchmarks constituting valuable tools for quantifying model performance in certain contexts, but not being suitable as the sole criterion for model selection or validation. The key points were:

- *The Need for Open-Source Resources:* The discussion underscored the importance of open-source tools and tutorials that make complex models accessible and comparable. Efforts at the Flatiron Institute to develop such resources, enhancing the accessibility and usability of computational tools in neuroscience, were particularly noted.
- *Benchmark Development for Neuroscience:* The idea of creating benchmarks, especially in areas that are not as well covered like motor function, was discussed as a means to enable standardized comparisons across studies. The panel discussed on the potential for such benchmarks to improve our understanding of the neural mechanisms underlying behavior and of model performance.
- *Model Complexity and Interpretability:* A significant part of the discussion revolved around balancing model complexity with interpretability. The conversation highlighted the tension between developing detailed models and the practicality of simpler models that still provide valuable insights. Broadly, it was acknowledged that models should be as simple as possible, but sufficiently complex to capture the nuances of neural data.
- *Supervised vs. Unsupervised Methods:* Participants reflected on the usefulness of both supervised and unsupervised analytical methods. While supervised methods allow for targeted analyses based on expected outcomes, unsupervised methods are crucial for exploring data without predefined categories or labels, offering a potentially broader understanding of neural dynamics.
- *Quality Assurance for Input Data:* The quality and reliability of input data were acknowledged as critical factors influencing the success of computational models in neuroscience. As a result, it was emphasized that thorough quality assurance and metadata richness are required to ensure that the data used in analyses accurately reflect the neural activity and behaviors being studied.
- *Community Effort and Collaboration:* Throughout the discussion, there was a strong emphasis on the importance of community effort and collaboration to improve research methodologies, develop benchmarks, and ensure data quality. Collaboration was recognized as essential for advancing the field of computational neuroscience and making meaningful progress in understanding the brain.

These key reflections highlight a collective vision towards a more integrated, accessible, and collaborative neuroscience research landscape, where computational models and benchmarks play a crucial role in advancing our understanding of the brain.

## IV. SYNTHESIS SESSIONS

The first two days of the symposium each ended with a “synthesis session”. During these synthesis sessions, participants came together to integrate and consolidate the diverse ideas, perspectives, and insights shared throughout the day. Synthesis sessions also served as a bridge between individual sessions, allowing for cross-pollination of knowledge and fostering a broader understanding of challenges ahead.

### A. Synthesis Session for Day 1 – Devices, Neuroinformatics, and Platform

The Day 1 Synthesis Session, moderated by Nima Dehghani (MIT) with panelists Mark Harnett (MIT) and Joseph Monaco (NIH Brain Initiative), was an in-depth discussion of the multifaceted challenges and opportunities in neurophysiology research, especially when it comes to data sharing, technological advancements, and research ethics. This summary synthesizes key points raised in the session, focusing on experience-based insights shared by participants within the overarching framework of seeking to advance neuroscientific research.

#### a. Technological Innovations and their Implications.

Participants reflected on the significant strides that have been made in neurotechnology, particularly with the development of new high-throughput acquisition devices. The discussion specifically underscored the ability of innovations in electrocorticography grids, volumetric recordings, and all-optical electrophysiology to critically improve both the spatial and temporal resolution of brain activity recordings, alleviating long-standing limitations in neuroscience research. These advances have the potential to enable groundbreaking discoveries and deepen our understanding of complex neural dynamics. However, with the ability of these technologies to improve experimental precision comes the challenges they pose in terms of data management and analysis.

#### b. Ethical Considerations and Animal Welfare.

A notable topic of discussion, unanimously recognized for its importance, was the ethical dimension of neurophysiology research, particularly concerning the use of animals. The dialogue underscored the importance of responsible research practices and the potential for open data to minimize animal use by maximizing the reuse of existing datasets.

#### c. Data Sharing, Metadata, and the Role of AI.

The session delved into challenges in data sharing and reuse, highlighting the critical role of comprehensive metadata in ensuring reusability and the challenges that arise when standardizing data formats. The potential pitfalls of metadata management and the paradoxical nature of data as both a boon and an obstacle to scientific progress were emphasized. The conversation also touched upon the role of AI and ML in enhancing data analysis and visualization, suggesting a future where AI could offer novel insights and improve efficiency in data handling.

#### d. Infrastructure, Support, and the Scientific Ecosystem.

Participants discussed the need for robust infrastructure and support systems to facilitate data sharing and collaboration. The conversation highlighted the challenges laboratories face when trying to adapt to rapidly evolving technologies and standards, and reinforced how access to centralized expertise or support teams could help alleviate this problem. The dialogue reflected a consensus on the need for sustainable models for conducting neuroscience research that enable scientists to focus on research questions rather than the technical nuances of data collection, analysis, and management.

#### e. Forward-looking Perspectives.

The synthesis session concluded with an acknowledgment of the persistent challenges in the field discussed above, but also a communal optimism when it comes to surmounting these obstacles through technological advancements, collaborative efforts, and supportive policies. The day’s discussions illuminated a path forward, characterized by a community-wide dedication to advancing neurophysiology research through open data, the pursuit of ethical practices, and the judicious integration of emerging technologies.

### B. Synthesis Session for Day 2 – Knowledge Extraction, Software, Modeling

The Day 2 Synthesis Session, moderated by Nima Dehghani (MIT) with panelists Matt Wilson (MIT), Ila Fiete (MIT) and Jim DiCarlo (MIT), ventured into the realms of collaborative efforts in data sharing, synergies between neuroscience and AI, and the difficulties that lie ahead for the analysis and interpretation of rich multimodal neurophysiological data. The session underscored the pivotal role of community, the imperative for developing advanced predictive models, and the necessity of creating robust educational and incentive frameworks to nurture a culture of innovation and openness in neuroscience.

#### a. Community and Data Sharing.

A strong emphasis was placed on the need for collaborative efforts within the neuroscience community to tackle the increasingly complex challenges that lie ahead. This includes the development and adoption of open data sharing platforms and scalable models that can support the future demands of the field. The session underscored the importance of creating and maintaining an infrastructure that fosters data sharing and collaborative research to accelerate scientific discovery and innovation.

#### b. Neuroscience and AI Integration.

Discussions delved into the interplay between neuroscience and AI, exploring how advancements in one field can propel the other forward. The conversation pointed out the current limitations faced by AI models when extrapolating beyond their limited training data, highlighting the unique insights that neuroscience can offer to improve AI models by drawing on the brain’s impressive generalization abilities. Conversely, AI’s potential to analyze vast datasets could unlock new knowledge in neuroscience, pointing to a symbiotic relationship between the two disciplines.

#### c. Data Models and Predictive Analysis.

A significant part of the conversation was dedicated to discussing how to develop models that better predict and simulate brain functions. Ideas included better integrating empirical data into theoretical models, thus enhancing the predictive power of these models. The need for benchmarks and standards to evaluate these models was also a point of focus.

#### d. Educational and Incentive Structures.

There was a call for enhancing educational resources to better equip researchers with the tools and knowledge necessary to navigate the growing complexity of neuroscience data and tools. Additionally, the discussion highlighted the need to rethink incentive structures within the scientific community to encourage data sharing, model development, and interdisciplinary collaboration.

#### e. Ethical Considerations and Data Quality.

Ethical considerations in data collection and sharing, particularly regarding human/patient consent and privacy, were raised. At the same time, concerns about the quality of data, and the importance of metadata and of standardizing data formats were brought up, with participants emphasizing the need for rigorous standards that ensure open data is both reliable and usable following FAIR (findable, accessible, interoperable, and reusable) standards.

#### f. Forward-looking Perspectives.

The session concluded with forward-looking statements about the future of neuroscience, pondering the types of technological advancements, community efforts, and theoretical breakthroughs needed to advance the field. The conversation touched on the prospects of building digital twins of the brain on which experimental questions could be tested, and the role of large-scale data analysis in understanding brain function at a deeper level.

## V. NEUROINFORMATICS BREAKOUTS

The third day of the symposium started with a keynote from a BRAIN Initiative representative. Following the keynote, attendees were divided into two focus groups. The first group concentrated on dissecting the challenges inherent to the field of neurophysiology, actively seeking and proposing solutions related to common infrastructure, data formats, and standards. This exploration aimed to identify and address the bottlenecks hindering data sharing and interoperability within the community. Meanwhile, the second group embarked on a deep dive into the transformative potential of AI/ML, computing, and visualization technologies in neuroinformatics. This group discussed how to leverage cutting-edge computational tools to enhance the analysis, interpretation, and visualization of complex neuroscience data, with the ultimate aim of accelerating discoveries and innovations in brain research. Through these parallel sessions, the symposium fostered a collaborative environment, encouraging the sharing of insights, experiences, and strategies to overcome the multifaceted challenges that face neurophysiology research.

**Beyond ‘FAIR’: What does sustainable protocolization of open data in neuroscience look like?** In his keynote for the breakout sessions, Joseph Monaco, a Scientific Program Manager in the Office of the BRAIN Director of NIH BRAIN Initiative, detailed the initiative’s strategic efforts towards establishing a sustainable and open data ecosystem in neuroscience. He revisited the BRAIN 2025 core principles, particularly emphasizing the commitment to establishing platforms for data sharing, with a focus on public, integrated repositories for datasets and analysis tools^73^. This approach is underpinned by a commitment to ethical standards in research involving both human and non-human subjects, and reflects a dual focus on innovation and responsibility.

Monaco elaborated on the envisioned BRAIN Data Ecosystem, describing a dynamic infrastructure designed to support data coordination, integration, interoperability, and reuse. This infrastructure aims to facilitate not only data sharing, but also replication studies, rigor studies, and secondary analyses for enhanced reproducibility and discovery. Central to this vision is the creation of a healthy, vibrant, multidisciplinary data ecosystem that aligns with open science principles, thereby accelerating the development and testing of theories and models of brain function. Monaco underscored the significance of the BRAIN Data Sharing Policy, which mandates regular submissions to the BRAIN data repositories, ensuring that data generated from BRAIN Initiative-funded research are made accessible to the wider research community in a timely manner.

Monaco also provided an overview of the diverse data domains within the BRAIN Initiative’s purview, including light microscopy, multi-omics, neurophysiology, human neuroimaging, spread across various repositories such as BossDB, NeMO, DANDI, OpenNeuro, and DABI (see Table II and III). These repositories collectively house thousands of datasets, demonstrating the vast scale and scope of neuroscience data being shared and analyzed already. Through this extensive data sharing and management policy, and through targeted funding opportunities and strategic mission goals, the BRAIN Initiative is fostering advances in data science and creating a robust infrastructure to ensure that research data can be widely leveraged to secure a deeper understanding of the brain, paving the way for groundbreaking discoveries in brain health and disease.

### A. Common Infrastructure, Data Formats & Standards

This breakout session, moderated by Dorota Jarecka (MIT), Billy Broderick (Flatiron Institute), Edoardo Balzani (Flatiron Institute) and Christian Horea (Dartmouth College), provided a detailed discourse on standardization and reproducibility. The session highlighted the ongoing challenges and evolving solutions to maintaining the integrity and verifiability of neurophysiology research as the use of large datasets and complex data analyses increases.

#### 1. Data Formats and Standards

The session started with a discussion of what the terms “data format” and “data standard” are commonly understood to mean, which was followed by an examination of the detailed definition of “data standard” provided by resources.data.gov. Reviewing the associated list of data standard components, i.e., datatype, identifiers, vocabulary, schema, format and API^74^, provided common ground for the subsequent discussion of the current status of and need for data standards within the community.

##### a. Ontology.

Discussions focused on how best to link descriptive terms used in neurophysiology with standardized ontologies (Ontologies provide a structured framework for organizing and connecting descriptive terms, facilitating better understanding and communication within the field). For instance, it can be very helpful to associate a brain area studied in an experiment with its defined region in a widely used brain atlas. Despite resources like the National Center for Biotechnology Information (NCBI) Taxonomy, Mouse Genome Informatics (MGI) database, and Neuroscience Information Framework (NIF) Standard Ontology being available, they are not always used by researchers due to the complexity and effort involved in entering precise and detailed metadata. To overcome these hurdles, participants in the breakout session proposed developing interfaces that provide default options or infer metadata from the data, simplifying the metadata identification and entry process. Participants also discussed the trade-off between strictly enforcing of accepted ontologies and maintaining flexibility even though the latter can decrease the data usability.

##### b. Data Standards.

Part of the discussion focused on the connection between schema, format, and API. The conversation emphasized the importance of not only storing data, but also structuring it in ways that facilitate access and interoperability. The flexibility of the NWB standard was highlighted, with debates around whether more components, such as the vocabulary used in metadata fields, storage formats, and APIs, should be standardized. The consensus leaned towards providing best practices and defaults rather than turning to stringent enforcement, to ensure the NWB standard can adapted to diverse research needs.

##### c. Improving Metadata Recording.

To promote the adoption of the NWB standard, it was proposed that data acquisition systems should be enabled to write raw data directly into NWB format. However, this direct conversion approach presents challenges, particularly regarding the comprehensiveness of metadata. Metadata includes information about data collection, experimental design, and subject details, all of which provide essential context for experimental data. However, many of these details are often collated after data acquisition begins. As a result, the NWB files created during acquisition may initially lack compliance with the standard’s own metadata requirements.

The session also highlighted the complexity of integrating multiple time-based data streams into a single NWB file, as this requires all data to be synchronized to the same clock. Given the diversity of experimental setups used in neuroscience, with data streams often operate on separate clocks, a potential solution discussed was to modify the NWB standard. This modification would allow the storage of raw data streams in their native clocks along with synchronizing pulse data, enabling post-hoc time alignment. This approach, however, would be hindered by the lack of universally accepted methods for sending synchronizing signals and performing time alignment across diverse systems.

Moreover, specific challenges arise with devices like the Neuropixels probe, where data from various channels are not sampled exactly simultaneously, but are instead slightly offset. The developers of SpikeGLX, commonly used with Neuropixels, recommend a method called “tshift” for aligning channels. It was therefore proposed that data acquisition systems initially store unaligned data in NWB format, allowing users or automated software tools to later apply tshift or similar methods to synchronize the channels. Further processing, like common average referencing or cross-stream alignment, could then be applied, with the processed data being cached either in the same NWB file or a new one for subsequent analysis.

Additionally, environmental metadata (e.g., temperature, humidity, luminance) is often omitted when recording experiment variables. To address this, NWB could introduce an optional schema for incorporating such environmental factors. Some of these metadata elements might even become mandatory as community practices evolve.

##### d. Data Curation.

Discussions also covered how experimental information — such as session or subject exclusions from analyses — is currently stored in laboratory notebooks or separate databases. Integrating this information into the NWB standard is crucial for fully understanding dataset usage and interpreting analysis results. However, since these annotations are highly specific and free-form, breakout participants debated best practices for including them in NWB files while maintaining flexibility to accommodate diverse experimental designs.

In summary, these discussions underscore the broader challenge of standardizing experimental metadata. Community-driven standards are essential to accommodate the complexities of neurophysiology research, striking a balance between strict standards and the flexibility needed for compatibility across various experimental designs.

#### 2. Common Infrastructure and Computational Reproducibility

The reliability of neurophysiology data processing and analysis is often jeopardized by the fact that software packages depend on specific environments and operating systems, both of which can vary widely in behavior and availability. Details as to which versions were used for software packages and their dependencies, and what environment they were installed in are seldom recorded alongside analysis results. This complicating the processes of reproducing results and of verifying how certain software bugs could affect data analysis outcomes.

##### a. Containerization as a Solution.

The potential of using containerization, through tools like Docker, to address these reproducibility challenges was discussed. Docker containers package an application with all of its dependencies into a single unit, ensuring it runs consistently across different computing environments. Writing a Dockerfile, which documents all commands necessary to build the application’s environment and install necessary packages, facilitates the process of containerization^75^. Using this approach, researchers to share not only their data but also the exact computational environment used to process that data, enhancing the reproducibility of scientific results.

##### b. Linking Containers with NWB.

The possibility of integrating Docker containers with NWB was considered as a way to further improve computational reproducibility. The NWB standard includes an optional “source-script” field, which could be used to store a Uniform Resource Identifier (URI) linking to a container image and the analysis scripts used, allowing other researchers to replicate the computational environment and analyses. However, NWB files often contain multiple processed data streams generated through different scripts, and NWB does not currently offer a standardized method for linking individual data streams to specific scripts or container URIs.

##### c. Debating Provenance Storage.

During the breakout session, participants engaged in discussions about where to store provenance information - specifically, details related to the inputs, settings, and outputs of computational analyses. The central question was whether this information should reside within NWB files or be managed externally. Provenance data play a crucial role in enabling users to understand the computational history of data files. When determining where and how such data should be stored in NWB files, an important consideration is how other standards and data management systems store provenance data. Notably, certain workflow engines and data frameworks already handle provenance information outside of NWB, e.g., ALPACA which stores the provenance data in Resource Description Framework (RDF) files^76^ or DataJoint which stores provenance data indirectly through a data processing pipeline backed by a database^77^. Thus, although these resources provide potential templates for how NWB could store provenance data, it might be more practical to instead leave the management of provenance data to these external resources.

##### d. Community Concerns and Realism.

It was recognized that while the technological solutions discussed above could significantly improve reproducibility, their widespread adoption by the neuroscience community might be hindered by lack of familiarity with and accessibility of containerization tools. Moreover, long-term archiving and costs associated with maintaining container images present additional hurdles that must be addressed to ensure these solutions are viable for the broader community.

### B. AI/ML, Computing & Visualization in Neurophysiology Research

Integrating AI and ML into neurophysiology research, particularly in the context of leveraging open data, comes with promise, but also challenges. In this breakout session, the discussion, moderated by Cody Baker (CatalystNeuro) and Guillame Viejo (Flatiron Institute), delved into strategies for harnessing the full potential of these technologies, while navigating the peculiarities of neurophysiology data.

#### a. Strategy.

To create a collaborative environment, the session began with participants specifying the point in the data lifecycle most relevant to their work. It was noted that the efficiency of computational performance is bound by existing infrastructure and data formats, which were focus points of the other breakout session. The discussion introduced the potential role of state-of-the-art large language models (LLMs), such as AmadeusGPT and BrainGPT, setting the stage for a comprehensive examination of AI/ML applications and their constraints in neurophysiology.

#### b. Morning Discussions.

The dialogue aimed to differentiate between AI and ML, defining AI as encompassing conceptual models, while ML encompasses practical tools. Participants in the breakout session also raised questions about the true advancement of AI beyond its application in sophisticated statistical learning. Distinguishing AI from ML tools is crucial for enhancing data sharing and discovery. The concept of an ‘AI-ready dataset’ emerged as a central theme—an adequately large and internally consistent dataset suitable for training and testing models. During the discussion, topics ranged from dimensionality reduction to the need for universal descriptions of computation types. Additionally, approaches for accurately aligning behavioral and neural data streams temporally were explored. Notably, the lack of standardized experimental protocols for time alignment posed a significant barrier, alongside challenges in effectively utilizing pre-configured rigs for new experiments.

#### c. Addressing Core Questions.

The conversation tackled several critical questions:

- *Reliance on LLMs for Data Analysis:* Whether the scientific community is ready to depend on large language models for critical tasks or reamins too skeptical due to the opaque nature of LLMs.
- *Balancing AI Tools with Mastery of Computational Skills:* Whether PhD students should focus on learning computational skills or rely on AI tools and specialists for efficiency.
- *Challenges for Neuroscience Researchers with Non-computational Backgrounds:* What emphasis should be placed on developing a solid understanding of the tools and techniques, to prevent misguided use of AI tools and incorrect interpretation of analysis results.

#### d. Identified Problems.

Two primary barriers to leveraging statistical learning methods in neurophysiology were pinpointed:

- *Experiment Diversity and Dataset Quality:* The significant diversity and heterogeneity of neurophysiology experiments pose a hurdle for ensuring data quality for meta-analyses, highlighting the need for ‘AI-ready datasets’. Additionally, difficulties in achieving precise temporal synchronization between behavioral events and neural data streams present a substantial challenge, and reflect the need for standardized practices.
- *Common Vocabulary for Neural Patterns:* The lack of agreement on a common vocabulary to describe neural patterns (such as ripples, bursts, avalanches, etc.) interferes with our ability to generalize statistical learning methods, and is a hindrance to community-wide communication.

#### e. Communication and Community Interaction.

The discussions underscored difficulties that arise when communicating about these nuanced topics, as laboratories worldwide have developed their own unique frameworks and terminologies centered on their own experimental setup designs and task protocols. Although this diversity may present certain advantages, it also reveals the need for enhanced community interaction and common ground to enable clear communication, bridge gaps in understanding, and enable a certain degree of standardization across the field.

#### f. Afternoon Insights.

Focus shifted towards the importance of establishing benchmarks and identifying data quality metrics to boost research reproducibility and reliability. Innovative strategies were proposed for enhancing overall data quality, like allowing external users to annotate datasets on DANDI, as well as derivative datasets (reprocessed from existing raw data sources). Suggestions like these reflect the need for collaborative approaches to overcome the multifaceted challenges that will arise as we move to better integrate AI/ML tools into neurophysiology research, including, but not limited to, community-driven efforts to establish common standards and improve data utility.

#### 1. Consistent Curation of Diverse Data

During the symposium, a consensus emerged that there is an abundance of machine learning models ready to meta-analyze data from repositories like the BRAIN Initiative’s DANDI repository. However, concerns were raised regarding the adequacy of available data annotations. The critical question was whether the data standards mandated by these repositories include sufficient provenance information to accurately describe the experimental session parameters^78^.

Creating and maintaining robust ‘AI-ready datasets’, of which prominent examples are MNIST for computer vision^79^ and GigaSpeech for speech recognition^80^, demands significant effort to ensure the accuracy of labeled features. The meticulous approach required to ensure this level of quality for neurophysiology data must contend with the typically high complexity of the data, which is often tailored to very specific experimental questions that may never be repeated closely enough across datasets. Although certain physiological datatypes, like fluorescence traces and spike trains, or behavioral metrics such as maze exploration trajectories, inferred running velocity on a rotating disc or ball, etc., are common to many experiments, they nonetheless require detailed descriptors to be fully understood. However, annotating behavioral data is often challenging. This highlights the need for tools and frameworks like the BAABL extension for NWB^81^ and Hierarchical Event Descriptors (HED)^82^ to standardize and improve data annotations for better reusability. Three primary reproducibility concerns were highlighted.

##### a. Hyperparameters and Metadata Documentation.

The neurophysiology community lacks consensus on the necessity of documenting all variables, including software versions involved in analyses (like spike sorting), to achieve identical results from the same raw data. It remains unclear what level of granularity is necessary for reliable reproducibility.

1. *Complexity of Models:* AI/ML models often rely on a myriad of hyperparameters that can dramatically influence their performance. Precisely documenting and replicating these hyperparameter settings is pivotal to ensuring results can be reproduced. Without such records, reproducing the exact behaviors of complex models becomes nearly impossible, leading to discrepancies in findings and interpretations across, and even within, research groups.
2. *Software Evolution*: The rapid evolution of software through continuous updates can introduce variability into analysis outcomes. Different versions of the same software can produce diverging results due to major, or even minor, alterations in the code underlying algorithms or processing techniques. Documenting software versions is thus essential for reproducibility, yet this practice is inconsistently applied across the community.
3. *Community Consensus*: The lack of consensus on whether highly granular reproducibility is needed reflects a broader challenge in the AI/ML and neurophysiology fields. While some argue that every aspect of an experimental setup and analysis should be detailed to ensure fidelity in replications, others view this level of detail as unnecessary or impractical, especially when considering the rapid pace at which technology and methodologies are advancing.
4. *Impact on Machine Learning Methods*: The effectiveness of AI/ML methods in learning from data and generalizing from their training datasets relies on the consistency and accuracy of the data and metadata they are trained on. Inconsistencies or omissions in model hyperparameters and metadata documentation can hinder the training process, potentially resulting in less effective models or, worse, models that perpetuate errors.
5. *Barriers to Collaboration and Innovation*: Inconsistent documentation practices not only hinder reproducibility, but also pose barriers to collaboration and innovation. Researchers attempting to build upon previous work may find it difficult to replicate studies accurately, slowing progress and potentially leading to a fragmentation of efforts across the field.

##### b. Documentation of Anomalies.

During experiments, non-protocol events (such as seizures, sneezing, or external disturbances) are seldom formally registered or manually recorded by the experimenter. If they are documented, it is often done informally, such as by jotting down a note in a laboratory notebook. Unfortunately, this information may not always find its way into the shared version of the data. This poses a challenge for data reusers, as these events, if not properly taken into account, can interfere with and bias analyses, including ML/AI analyses. The discussion underscored a significant concern: without the ability to effectively filter out data affected by anomalous events, ML/AI methods might mistakenly identify them as significant and, if these events are included in algorithm training datasets, this could lead to biases in how these algorithms are then applied to standard data. This problem underscores the importance of developing assistive tools for consistently annotating data on the fly, and machine learning tools with robust filtering capabilities that can distinguish between typical experimental data and anomalies arising from unforeseen events.

##### c. Enhancement of Data Quality through Quality Metrics.

To enhance data quality in neurophysiology repositories, a suite of quality metrics is proposed. Taking inspiration from the MRIQC package for MRI data^83^, the goal is to create a common software package that allows real-time quality assessment of data during experiments. Researchers, while conducting experiments, can utilize this tool to assess data quality promptly. By establishing agreed-upon quality metrics across various modalities, we can significantly improve users’ ability to identify high-quality datasets, leading to more reliable and interpretable scientific findings.

#### 2. Temporal Alignment of Neural and Behavioral Streams

Neurophysiology experiments characteristically involve simultaneous acquisition of neural recordings and tracking of behavior. The importance of precise synchronization between these data streams cannot be overstated, especially considering the rapid timescales at which neural activity evolves. Traditionally, laboratories have addressed this requirement through two main approaches: developing customized in-house rigs tailored to their specific experiments, or investing in pre-fabricated setups purchased from specialized manufacturers. While pre-fabricated setups offer convenience and standardization, they often come with high costs and potential vendor lock-in, limiting flexibility for integrating additional data streams. In contrast, custom in-house rigs, despite their potential for tailored experimental design, introduce a significant risk of errors. These can stem from a lack of standardization or insufficient technical expertise, both of which can compromise data integrity and, thus, the validity of experimental outcomes.

Overall, it can be very challenging to balance customization with reliability in experimental setups. However, even when a laboratory converges on reliable and sufficiently customized experimental setups, the notable scarcity of educational resources or comprehensive guides, and the high turnover rate within laboratories can lead to serious knowledge transfer failures. Altogether, these obstacles make it difficult to ensure that complex and sensitive tasks like precise temporal alignment are correctly performed. They also make it challenging for the open science community to develop widely applicable solutions to shared problems like the temporal alignment problem.

##### a. Proposed Solutions.

To mitigate these challenges, it was proposed that a public repository for documenting experimental protocols be created. This repository would serve as a centralized resource for finding detailed protocols, like those pertaining to correct temporal alignment across data streams. Currently, the granularity of method sections in publications is often insufficient for replicating or adapting experimental designs. Direct communication with the original researchers is then required, even though it is likely to be time-consuming on both ends and inefficient. However, in some cases, the information is simply unavailable. If the person who conducted the experiments is no longer accessible, and knowledge transfer (KT) was not adequately done, critical details about the experimental setup may be lost. Contributing detailed instructions to a communal repository could significantly enhance inter-laboratory communication, streamline the design process for new experiments, and ensure a higher degree of reproducibility across the field. By providing a path to standardizing and validating these protocols, such a repository would facilitate knowledge sharing, while also supporting the creative diversity essential for scientific innovation.

##### b. Balancing Learning with Efficiency.

While there has been a push to streamline experimental setup designs through standardized protocols, it is also important to recognize the value of experiential learning for students, postdocs and junior researchers. Engaging deeply with the process of designing and implementing neurophysiology experiments, from conceptualization to the intricate work of setting up equipment, can set early-career researchers up for success by solidifying their grasp of the technology used in their research. This hands-on experience is crucial for ensuring each new generation of scientists are generally able to troubleshoot their own experiments and innovate within the complex landscape of neurophysiology research. In conclusion, while standardized documentation and shared protocols can significantly reduce entry barriers and enhance experimental reproducibility, they must complement, rather than replace, the invaluable learning that comes from direct engagement with experimental design and execution.

#### 3. Vocabulary of Neural Patterns

Progress has been made in standardizing how behavior is represented and recorded in neurophysiology experiments. Yet, consensus is elusive regarding how to ensure that specific neural activity patterns are characterized and identified in a consistent manner across different experimental conditions. This area of study includes, for example, classifying cell types based on waveforms from spiking events^84–86^ and identifying graphoelements such as hippocampal sharp-wave ripples or cortical spindles, which are observed over time, in response to stimuli, or as a result of intricate thalamocortical interactions. Typically, these types of neural activity are categorized through visual inspection and expert judgment, often supplemented by contextual information like knowledge of the subject’s engagement in a task or state of consciousness. However, it is debated whether these subjective methods of classification are sufficiently consistent and rigorous to accurately describing neural patterns. A major challenge to automating the process of quantifying graphoelements is the lack of consensus among experts about the key spatiotemporal characteristics of elements like sharp-wave ripples^87,88^. More standardized definitions and methodologies for labeling these neural patterns are needed to ensure that research findings in the field are clearly interpretable and reproducible. Successful examples of automatic detection and characterization of complex spatiotemporal graphoelements include the use of sequential spectral density methods or neural networks for the characterization of spindles^89,90^, and deep learning for the detection of sharp-wave ripples^54^.

Improving the specificity and consistency of data labeling, particularly for datasets involving experimentally introduced stimuli, presents a unique challenge. One proposal is to sequester certain data segments within public datasets for server-side verification purposes, a technique commonly employed in ML/AI competitions. This approach would balance the importance of making all original experimental data open access for reproducibility and reuse, with the value of reserving some data for assessing and validating derivative ML/AI algorithms.

Considerations like these reveal a broader issue: the fact that specific assumptions are made when interpreting data for individual studies, and that this in turn influences how datasets are curated and can subsequently be reused. A move towards universally accepted metrics and labeling conventions could substantially benefit the scientific community, ensuring datasets preserve a wider relevance when shared, and allowing for more straightforward cross-study comparisons and integration.

Recognizing the need for greater standardization, initiatives like the Brain Behavior Quantification Synchronization (BBQS) have been launched by funding agencies, including the NIH. These efforts aim to establish clearer guidelines and tools for documenting and analyzing behavioral and neural data, ultimately fostering a more cohesive and collaborative research environment.

## VI. A FORWARD LOOKING PERSPECTIVE

### A. Building Communities

#### a. Building a Community.

Open science thrives in a well-supported ecosystem where community-based governance and communication can flourish^7^. The nascent ODIN community will require robust mechanisms for dialogue and self-regulation, ideally emerging organically from within the community itself. An prime example of this model is Wikipedia, which thrives under self-imposed rules and a transparent decision-making process. Unlike transient tools like team communication platforms, a wiki provides a durable, public, and cumulative resource for community discourse^91^. Engaging in quality discussions and integrating these alongside the data itself will ensure accessibility and transparency for the wider public.

#### b. Annual Meetings.

The enthusiasm shared during this symposium suggests a strong desire for it to continue annually. These meetings are envisioned as key catalysts for fostering a robust ODIN community, and drawing together diverse voices from across the neurophysiology and systems neuroscience spectrum. By maintaining open communication channels and featuring varied perspectives, we hope to enrich our collective knowledge. In addition, we hope that continuing to share these talks on widely-used and open video sharing platforms will ensure broad, public accessibility and engagement.

### B. Harnessing Large Language Models (LLMs)

The advent of advanced LLMs such as *OntoGPT*^92^ and *BrainGPT*^93^ heralds a transformative shift in how scientific information can be processed, understood, and utilized. These models have demonstrated a remarkable ability to distill and predict complex patterns from vast datasets, suggesting a potential role in enhancing user interaction with neuroscientific databases. AmadeusGPT showcases an innovative application in this direction, using LLMs to convert natural language descriptions of animal behaviors into executable analysis code, thereby facilitating interactive behavioral research, and increasing its accessibility^94^. Tools like these exemplify the potential of LLMs to help bridge gaps between complex biological knowledge and expertise in computational analysis, enhancing scientists’ ability to access and analyze neuroscience data in their research. Discussions at the symposium also touched upon how LLMs could help researchers engage more effectively with existing scientific knowledge, for example through enhanced literature searches and dynamic knowledge base augmentation via scientific journal content distillation.

For example, BrainGPT has been specifically trained to anticipate the outcomes of neuroscience experiments by ingesting extensive portions of the neuroscientific literature. Its proficiency, as demonstrated by the BrainBench benchmark, surpasses that of human experts in distinguishing between true experiment results and modified abstracts^93^. Capabilities like these suggest a future where LLMs could be used to reliably navigate and summarize existing scientific knowledge. It is important to note, however, that LLMs are subject to hallucinations. For this reason, BrainGPT is not currently enabled to perform this type of task^95^, and this potential use remains a matter of conjecture for the moment. Likewise, another potential use for LLMs is in inductive reasoning^96^. In the case of open data, an LLM trained in this way could be harnessed for hypothesis generation and experiment planning.

OntoGPT’s approach to enhancing knowledge bases through natural language processing highlights another very promissing application for LLMs^92^. By helping construct and refine knowledge bases, LLMs can facilitate more accurate and dynamic querying of complex data structures. When it comes to managing extensive open neuroscientific data repositories, integrating LLMs could dramatically improve the precision and scope of data retrieval processes, enabling researchers to generate interconnected insights from disparate datasets.

#### a. Practical Applications of LLMs in open neuroscience.

- **Enhanced Literature Search:** LLMs could be utilized to refine literature search mechanisms, enabling researchers to rapidly locate relevant studies and datasets. By processing queries using LLMs trained on the latest research and reviews, we could offer more contextually aware search results, reducing the time spent on literature reviews and increasing the relevance of the information retrieved.
- **Knowledge Base Augmentation:** Using LLMs like OntoGPT to assist in the ongoing development and expansion of neuroscientific knowledge bases could help ensure that data curation and query management follow standard nomenclature and are consistent with open neuroscience practices. For example, LLMs can assist in linking new data entries with existing ontologies and suggest updates to improve the comprehensiveness and utility of a database.

#### b. Future Directions.

As LLMs evolve, we should be able to leverage them not only to manage and query existing data, but also to anticipate and prepare for future research developments. Continuous updates to LLM training sets and algorithms and fine-tuning, using methods like LoRA^97^ and Retrieval Augmented Generation (RAG)^98^ techniques, will be essential to maintaining their effectiveness and relevance to the neuroscientific context. Overall, incorporating LLMs into open data in neuroscience promises to enhance utility as a dynamic, forward-looking tool that not only serves current user needs, but also adapts to and anticipates future scientific challenges.

### C. Addressing Community Needs

For open science to advance in neuroscience, it is critical to understand and address the needs of the community. This includes continuing to develop resources and tools that make open science practices easier to adopt for users of all backgrounds, while also ensuring that funding and incentives are in place to enable users to invest the time and effort that is still required. Table IV summarizes key community needs identified during the symposium.

### D. Recommendations for the Practicing Neuroscientist

As we continue to innovate and advocate for community-wide advancements in open science, practicing neuroscientists have several opportunities to engage with the existing open science practices. This section makes recommendations that span the entire lifespan of a project and can greatly improve the reproducibility and efficiency of one’s research. Depending on their projects and access to resources, individual research groups may find certain recommendations more relevant, helpful or feasible to implement than others. We recommend identifying these priorities and approaching the adoption of open science practices incrementally.

#### a. Data Management and Sharing Plan.

**FIG. 2:**
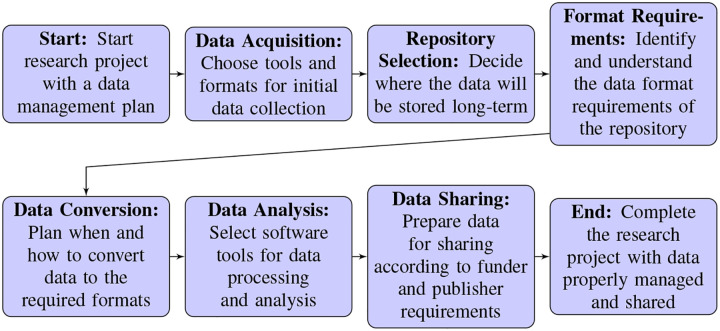
Data management plan flowchart.

It is important to prepare a data management and sharing plan early in the research process. Funders like the NIH and many scientific journals now require open sharing of data collected under their grants and for publication, respectively. Deciding early on which repository to use, understanding its data format requirements, and planning the workflow from data acquisition to publication can greatly facilitate the process. Adopting standards such as NWB early in data acquisition can also streamline the process and save time by ensuring consistency is maintained through data processing, analysis, and publication. Steps to follow are (see Fig. 2):

1. Identify the repository where your data will be deposited.
2. Understand the data format requirements of the chosen repository. Determine whether your data needs to conform to specific standards such as NWB.
3. Plan how you will manage, process, analyze, and visualize your data:
  - What software tools will you use?
  - What data formats do these tools accept, and what formats do they output?
4. Plan for and implement the use of common data standards as early as possible in the data lifecycle: from acquisition through processing, analysis, and up to publication and sharing. Early adoption streamlines workflows, avoids the need to refactor custom code down the road, and enhances the reusability of data, saving time and resources.
5. Standardize the process of converting your data into the required standard format. If done at the acquisition step, make sure that the proper metadata is included. If certain post-processing steps are required routines in your laboratory, make sure to track the details of how they were run and include the relevant information during data conversion to standardized formats.

#### b. Documentation and Metadata.

**FIG. 3:**
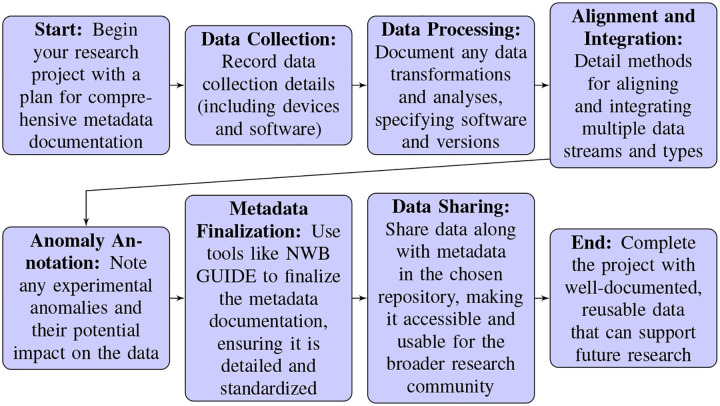
Documentation/Metadata flowchart.

- **Detailed Documentation:** Providing thorough and structured metadata is essential for enabling effective use of your data and reuse by researchers who are not familiar with your project. When storing and sharing data, aim to (see Fig. 3):
  1. Document the **source script** and any other processes used to generate the dataset, even if they are not mandatory fields in your chosen data standard.
  2. Include comprehensive details about the **devices, software versions**, and **analysis algorithms** used during the experiments.
  3. Record any **stimuli** presented during the experiments, and include a detailed table specifying which stimuli were presented when.
  4. Clearly describe how **neural, behavioral and stimulus data streams** were aligned temporally.
  5. Record key subject descriptors, like **genotype**, referencing external databases for standard definitions where applicable.
  6. Annotate any **anomalies or unusual occurrences** during data collection that might affect subsequent analyses.
- Utilize tools like **NWB GUIDE** for user-friendly and automated capture of important metadata to minimize effort and enhance standardization.
- As AI/ML methods become increasingly integrated into neurophysiology data analysis, ensuring your datasets are **AI-ready** with rich metadata will greatly enhance their reuse and the reliability of subsequent findings.

#### c. Utilizing Existing Tools.

**FIG. 4:**
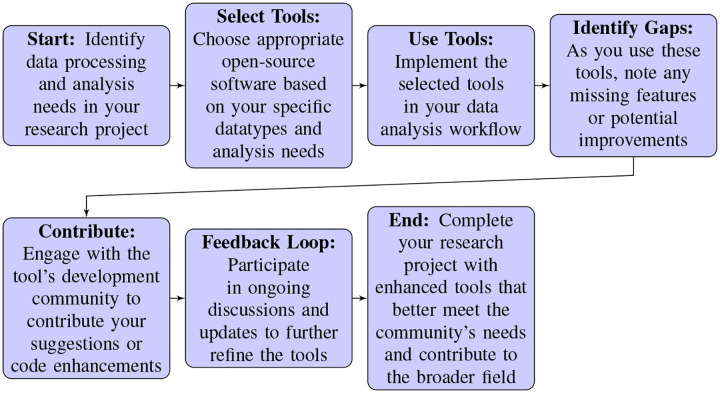
Tooling flowchart.

Several steps can help optimize your use of existing tools (see Fig. 4):

- **Choosing Open-Source Software:** Due to the complexity of neurophysiological data analysis, it is advisable to use established open-source software packages, when applicable. These are less prone to errors and are continually vetted by the community. Examples include:
  1. **Spike Sorting and Processing:** Consider tools like SpikeInterface and KiloSort.
  2. **Calcium Imaging Data Processing:** Consider tools like suite2p and CaImAn.
  3. **Pose Estimation:** Consider tools such as DeepLabCut and SLEAP.
- **Contributing to Tool Development:** If existing tools lack certain features or could be improved, contribute your enhancements back to the project. This type of collaboration:
  - Allows the community to verify the robustness of the new feature.
  - Enhances tool functionality and utility for the entire community.
  - Accelerates scientific discovery and increases the robustness of research outcomes.
  - Builds a culture of reuse and improvement, aligning with open science principles.

#### d. Developing New Tools.

If you develop new tools from scratch, it can be very valuable to share these with the broader community. To maximize the robustness, usability, and findability of these tools, it is particularly helpful to:

- **Share** the code on a platform like GitHub that enables robust version-control, as well as user feedback and contributions, ideally under a license that is highly permissive for code reuse and adaptation.
- **Document** the code by including at minimum a README explaining the tool’s intended use, the programming language it is designed in, its dependencies, usage examples, and ideally an interactive tutorial users can run in the cloud. More detailed recommendations can be found in previously published articles^99^.
- Make a **plan** for long-term maintenance and promotion of the tool. This may require investing financial resources and hiring of dedicated personnel, but is generally critical for the longevity and usability of an open-source tool.

## VII. CONCLUDING REMARKS

**FIG. 5:**
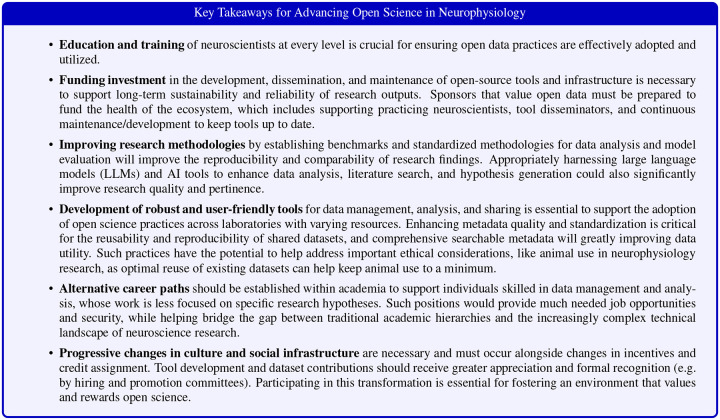
Key takeaways.

This first ODIN symposium highlighted a growing momentum in neurophysiology research to incorporate the values and practices of open science. The key takeaways from the symposium are summarized in Fig. 5. Innovations revolutionizing the quality and quantity of data we collect have been complemented by the development of robust data standardization and sharing platforms, along with a variety of computational resources for data processing, analysis and visualization. Large-scale data collection efforts are tackling the challenges of reproducibility and reliability in the field, with centralized approaches providing access to high quality data collection pipelines and decentralized ones encouraging collaborative protocol and analysis designs. However, significant challenges remain, particularly for laboratories with limited resources, where incorporating open science practices can be daunting and time-consuming. Furthermore, when laboratories do invest in adopting these practices, the time and effort required are often not sufficiently recognized by the traditional incentive structures of academic research.

Funding sponsors, publishers, and institutions wield the power to drive collaborative progress and sustain momentum. Their active support and recognition of researchers’ time and effort invested in open science initiatives are crucial for enabling this pivotal change in modern neuroscience. By acknowledging the value of open science practices, they elevate the entire field. Through incentivizing open data practices, funding robust infrastructure, and promoting tool dissemination, they create an environment where open science becomes a central pillar of neurophysiology research.

Meanwhile, we encourage researchers to actively engage in open science practices and leverage existing resources. By participating in the communities that build and use advanced tools, individuals can discover solutions to challenges they face, and tap into valuable community support. When solutions are lacking, researchers can provide feedback reflecting their specific research needs, increasing the likelihood that future iterations will address those needs. Thus, while transformative impact arises from collective action with the much needed support of funders and institutions, it is essential to recognize the power of individual voices in shaping this action.

Overall, we anticipate gradual, collaborative progress in the field, rather than an overnight transformation, engaging researchers, sponsors and institutions. We advocate for acknowledging and celebrating symbiotic developments, which together will propel us toward more open, transparent, and impactful science. In this context, the ODIN symposium (intended as a bi-annual event) can serve as a vital platform for sustaining momentum, sharing novel developments, and addressing the evolving needs of the community.

## Figures and Tables

**TABLE I:** Neuroscience toolkits presented or discussed at ODIN 2023.

Resource	Website	Tags
DANDI Archive	https://dandiarchive.org/	data repository
EB RAINS	https://search.kg.ebrains.eu	dataset search, knowledge graph, web app
DataJoint	https://datajoint.com/	data management, database, SQL, Python
SpyGlass	https://github.com/LorenFrankLab/spyglass	data management, database. Python
Dendro	https://github.com/flatironinstitute/dendro	cloud computing, web app
Neurosift	https://neurosift.app	visualization, dataset exploration, web app
NWB GUIDE	https://github.com/NeurodataWithoutBorders/nwb-guide	data format conversion, desktop app
NeuroConv	https://github.com/catalystneuro/neuroconv	data format conversion. Python
Neo	https://github.com/NeuralEnsemble/python-neo	data format reading. Python
Spikelnterface	https://github.com/SpikeInterface/spikeinterface	spike sorting, electrophysiology. Python
rippl-AI	https://github.com/PridaLab/rippl-AI	SWR detection, electrophysiology. Python
OptiNiSt	https://github.com/oist/optinist	ROI segmentation, optical physiology, desktop app
Caiman	https://github.com/flatironinstitute/CaImAn	ROI segmentation, optical physiology. Python
EXTRACT	https://github.com/schnitzer-lab/EXTRACT-public	ROI segmentation, optical physiology, MATLAB
suite2p	https://github.com/MouseLand/suite2p	ROI segmentation, optical physiology. Python
DeepLabCut	https://github.com/DeepLabCut/DeepLabCut	pose estimation, behavior. Python & desktop app
Lightning Pose	https://github.com/danbider/lightning-pose	pose estimation, behavior. Python
SLEAP	https://github.com/talmolab/sleap	pose estimation, behavior. Python & desktop app
VAME	https://github.com/LINCellularNeuroscience/VAME	pose estimation, behavior. Python
MoSeq	https://github.com/dattalab/moseq2-app	video sequencing, behavior. Python
CEBRA	https://github.com/AdaptiveMotorControlLab/CEBRA	data analysis, latent space, behavior. Python
Pynapple	https://github.com/pynapple-org/pynapple	data analysis. Python

**TABLE II:** BRAIN Initiative data archives.

Archive	Link	Datatypes	Access Restrictions
**BIL** (**B**rain **I**maging **L**ibrary)	https://www.brainimagelibrary.org/	Confocal microscopy brain imaging	Some restricted datasets
**bossDB** (**B**lock and **O**bject **S**torage **S**ervice **D**atabase)	https://bossdb.org/	Electron microscopy and x-ray microtomography	Public
**DABI** (**D**ata **A**rchive for the **B**RAIN **I**nitiative)	https://dabi.loni.usc.edu/	Invasive human neurophysiology	Some restricted datasets and requires registration
**DANDI** (**D**istributed **A**rchives for **N**europhysiology **D**ata **I**ntegration)	https://www.dandiarchive.org/	Cellular, systems, and behavioral neurophysiology	Public
**NEMAR** (**N**euro**e**lectro**m**agnetic **D**ata **A**rchive and **T**ools **R**esource)	https://nemar.org/	Electroencephalogram (EEC) and magnetoencephalography (MEG)	Public
**NeMO** (**N**euroscience **M**ulti-**O**mic **D**ata **A**rchive)	https://nemoarchive.org/	Multi-omics	Some restricted datasets
**OpenNeuro**	https://openneuro.org/	Magnetic resonance imaging (MRI) and other types of neuroimaging	Public

These archives are generally public access, although some house restricted datasets. Mostof these archives also allow embargoes, i.e., restricted access for a fixed period of time after initial publication.

**TABLE III:** Generic archives that contain some neurophysiology data. All of these are public access.

Archive	Link	Datatype
**Brain/MINDS Data Portal** (Japan’s Brain Mapping Project)	https://dataportal.brainminds.jp/	Includes marmoset structural and functional physiological data
**CRCNS** (**C**ollaborative **R**esearch in **C**omputational **N**euroscience)	https://crcns.org/	Neurophysiology data
**Dryad**	https://datadryad.org/	General research data
**EBRAINS**	https://ebrains.eu/	Various types of neuroscience data
**Figshare**	https://flgshare.com/	General research data
**G-NODE** (**G**erman Neuroinformatics **Node)**	https://gin.g-node.org/	Neurophysiology data
**Zenodo**	https://zenodo.org/	General research data

**TABLE IV:** Community needs and actions for advancing open science.

Category	Actions	Key Concepts
Guidance	Provide community guidance on sharing methodologies, datatypes (raw, processed) Standardize required and recommended metadata types Select and unify ontologies for metadata standardization, Define essential provenance information for shared data.	Provenance, shared methodologies, standardized metadata, unified ontologies
Tool Development	Enhance tools for data compression, conversion, sharing, and analysis. Develop cloud-based data access and analysis solutions. Establish benchmarking platforms for model and theory evaluation. Develop platforms for tool comparison. Support large-scale data pooling and annotation. Simplify metadata entry through user-friendly interfaces. Improve automated metadata capture tools. Enable on-the-fly data annotation of anomalies during experiments. Improve ability to detect and filter anomalous data.	Cloud solutions, data compression, data pooling, metadata entry, tool benchmarking
Research	Improve models for understanding complex data. Create benchmarks and metrics for model evaluation. Develop data quality assurance metrics. Innovate automated data labeling for enhanced data reuse.	Advanced model zoo, automated data labeling, data quality metrics, model benchmarks
Databases	Maintain centralized databases for datasets, methodologies, and tools. Facilitate community feedback mechanisms for shared resources.	Centralized databases
Knowledge Graphs	Create knowledge graphs for describing entities and their relationships, and for linking disparate databases.	Knowledge graphs
Education	Continue to develop online resources and training for data processing and analysis tools.	Online resources, training workshops
Funding & Incentives	Support community engagement and multi-laboratory collaborations. Fund technical personnel for open-source software maintenance. Encourage and facilitate adoption of new technologies and open science practices. Invest in scaling data storage solutions.	Community engagement, multi-laboratory collaboration, open-source support
